# Incisor Disorders of Merino Sheep (*Ovis aries*)

**DOI:** 10.1177/08987564251339058

**Published:** 2025-05-08

**Authors:** Alexandra Holt, Fritha Langford, Ankush Prashar, Helen Rogers

**Affiliations:** 1 Newcastle University, School of Natural and Environmental Science, Newcastle upon Tyne, UK; 2 Newcastle University, School of Dental Sciences, Newcastle upon Tyne, UK

**Keywords:** incisors, dental disorders, sheep, Merino, dental anomalies

## Abstract

This study documents dental anomalies in 2414 Merino ewe sheep (*Ovis aries*) from 7 Australian Merino Sire Evaluation Association sites observed during routine husbandry. Teeth were photographed and evaluated using visual scoring protocols to assess plaque accumulation, enamel wear, and other anomalies across all incisors. Plaque scoring revealed a median plaque score of 3 (IQR ± 1) on buccal surfaces and 2 (IQR ± 2) on lingual surfaces, indicating substantial plaque accumulation across incisors. Two cases of geminated incisors and one case of fused central incisors were identified, representing the first documented cases in sheep and expanding existing knowledge of dental anomalies in sheep. Dental wear assessments showed that 13% of 10-month-old ewes and 33% of 20-month-old ewes had significant enamel loss or pulp exposure, with an average of 2 teeth per affected ewe displaying pulp necrosis. Amelogenesis imperfecta was recorded in 5% of the younger and 37% of the older groups. Missing incisors, suggesting early dentition changeover, were observed in 9% of 20-month-old sheep. Localized enamel hypoplasia, potentially resulting from trauma or disruptions during enamel development, was noted in 1% of both age groups. Dental caries was identified in five 20-month-old ewes with permanent incisors. These findings provide a comprehensive overview of the range and prevalence of dental conditions in Merino sheep, including common wear patterns and rare anomalies. Documenting such conditions in sheep contributes valuable information to veterinary dentistry, highlighting the need for further research into incisor health in sheep.

## Introduction

Sheep, as grazing ruminants, rely on a functional incisor apparatus for successful foraging in diverse and often challenging environments. The loss of incisor function can severely affect a sheep's ability to grasp pasture, efficiently masticate, and adequately ruminate.^1–3^ Sheep are considered “low grazers,” preferring to graze at ground level and continuing even when plants are short and close to the soil, which makes them particularly susceptible to incisor dysfunction.^4^ Research has shown that sheep with incisor loss consume nearly half as much per bite as those without incisor issues, restricting critical fodder prehension ability.^5,6^

Incisor disorders and abnormalities can result in incisor loss or dysfunction, impacting overall health and productivity with reduced body weight,^6–9^ fiber production,^10^ and lower lamb-rearing capabilities.^7^ Ewes with missing incisors in lactation will experience impeded rates of repletion of skeletal calcium from the annual lactation cycle, affecting milk production and causing more incisor loss.^6^ Australian ewes with missing incisors were reported to have finished lactation approximately 6 kg lighter in body weight than those without missing incisors due to possible lower food consumption.^11^ Lambs from affected ewes were weaned at lighter weights, suggesting reduced milk production.^11^ A study of wool production from 7-year-old Merino castrates showed that production was greater in sheep with all incisors than those missing incisors.^12^ Similar associations have been found in Australian Angora goats, which showed a substantial decline in mohair production and staple length associated with 30% wear of permanent incisors, likely due to reduced food intake.^13^

**Figure 1.**
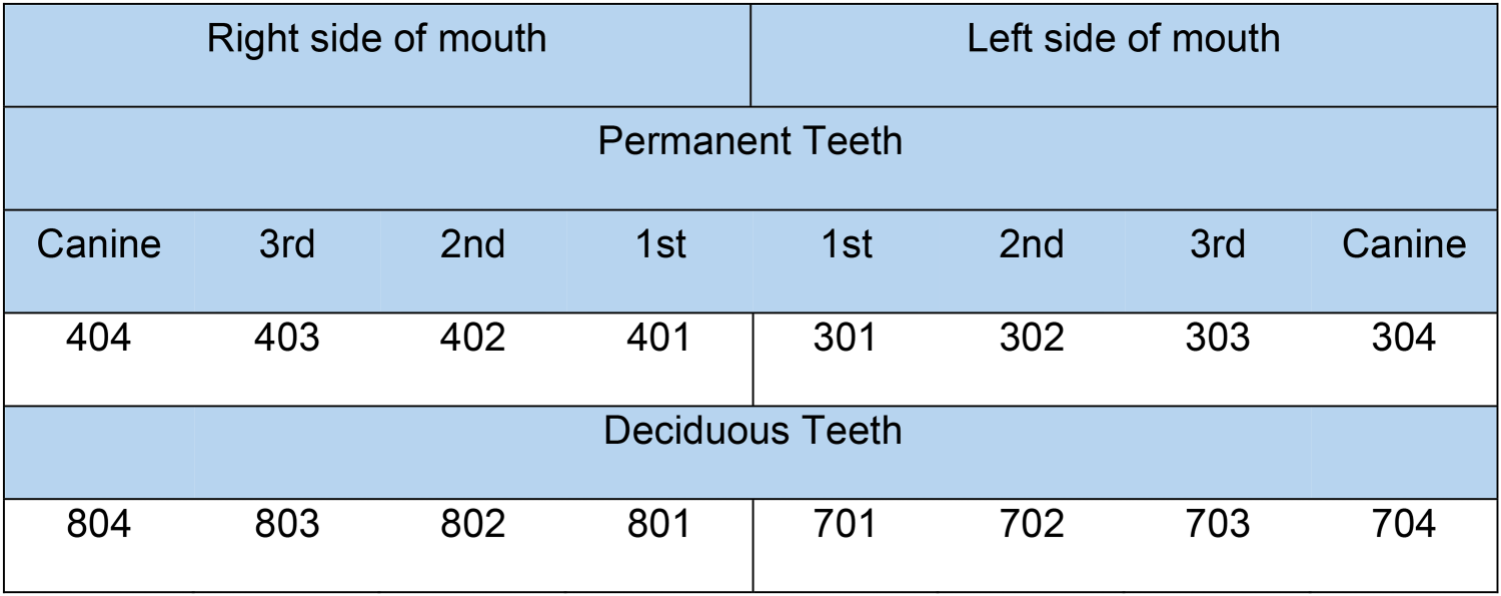
Modified Triadan numbering system for the sheep's mandibular incisor teeth.

Understanding the normal dental anatomy and physiology of sheep is critical for diagnosing, managing, and potentially preventing incisor wear, tooth loss, and periodontal disease. Sheep have 8 mandibular incisors, with the maxillary incisors being absent and instead, there is a dental pad of fibrous tissue covered by keratinized epithelium.^14^ The 2 most distal mandibular incisor teeth are technically canine teeth but functionally and morphologically resemble incisors and are generally referred to as incisors.^15^ Sheep are born with a relatively advanced set of deciduous teeth that, at 4 weeks, are fully functional.^16^ From approximately 12 months of age, sheep shed their deciduous teeth which are then replaced by permanent teeth. The Modified Triadan System was used to identify incisors^17^ (Figure 1). Between 12 and 14 months, the left (301) and right (401) first incisors (central incisors) erupt. The left (302) and right (402) second incisors erupt between 18 and 26 months, and the left (303) and right (403) third incisors erupt between 24 and 36 months. By 32 to 48 months, the fourth pair, the left (304) and right (404) canines erupt. After 40 months of age, all permanent incisors should have fully erupted. Sheep are referred to as “full mouth” when the 6 permanent incisor teeth erupt, as canine eruption has been observed to vary by as much as 8 months and not erupt in up to 8% of sheep.^16,18^

After 40 months of age, all 8 permanent incisors should have fully erupted. Sheep dental disorders, encompassing abnormalities and diseases, may be due to genetics, environmental factors, or a combination. Dental disorders and anomalies have been described in many animals, including dogs,^19–22^ horses,^23–25^ goats,^26–29^ and cattle.^30^ Research on sheep dental disorders has historically focused on 2 main incisor conditions—periodontitis and excessive wear. These 2 issues have often been studied independently despite literature indicating a potential interrelationship between them and other dental disorders.^31^ Due to the extensive research on both periodontitis and excessive wear, they are frequently cited as the most prevalent dental disorders affecting sheep worldwide.^31–36^ However, it is essential to recognize that current literature may have overlooked other incisor disorders that have not been rigorously explored. Excessive wear is a commonly used term in previous studies on sheep, suggesting that only severe cases are problematic. However, it is acknowledged that tooth wear is a multifactorial condition, leading to pathological dental hard tissue loss with several signs and symptoms, which may be detrimental to function.^37^ This study aimed to comprehensively assess the prevalence of incisor disorders in sheep, providing insights that may inform better management and prevention strategies.

## Materials and Methods

The research population comprised 2414 sheep on 7 Australian farms. All sheep were the progeny of the Australian Merino Sire Evaluation Association. All sites were visited in 2023. Site 1 was comprised of 434 Dohne Merino ewes aged 20 months with 2 permanent teeth. Sites 2 to 7 were comprised of 1980 Merino ewes aged 10 months with deciduous incisors and an average site size of 330 ewes.

All examinations of sheep incisors were undertaken as part of research approved by Newcastle University Animal Welfare Ethical Review Body AWERB Project ID No: ID 1047.

All study data were collected between May and December 2023. Each ewe underwent examination on their home farm premises. The incisor assessment was carried out during a routine handling procedure on the farm to ensure the least intrusive and minimally aversive handling. Each sheep was restrained upright using a VeeEzy sheep conveyor handler^a^, with each assessment lasting approximately 60 seconds. The sheep's mouth was held open, and an intraoral dental camera^b^ was used to take images of the buccal and lingual surfaces of the incisors. The study population exhibited typical ovine dental anatomy, with 8 permanent incisors at the mandibular arcade's rostral end, opposing the dental pad. Four photographs of all incisors from each sheep were captured. After the assessment, each sheep was released into a holding pen for husbandry to be undertaken according to the farm's standard practice.

The assessment was undertaken on the computer using photographs to minimize the time spent on animal restraint while providing a detailed view of the incisors. Incisors were assessed for missing incisors, plaque, caries, wear, and any anomalies. The photographs and assessments were reviewed by the same individuals (AH/HR). Various anomalies, including twinning anomalies, gemination and fusion, and localized enamel hypoplasia, were identified using visual assessment methods based on appearance.

This study employed visual phenotype classification to assess the likelihood of Amelogenesis imperfecta.^38^ However, the study did not conduct radiographic imaging, detailed histological analysis, or sensitivity assessments. As a result, the diagnosis is considered likely but not definitive, reflecting the reliance on visual assessment alone.

Missing or lost incisors were defined as incisors without observable evidence of permanent successors emerging. Due to the age of the sheep in the current study, the usual diagnosis of periodontitis was not applied; previous sheep research considers periodontitis a straightforward diagnosis when permanent incisors have been lost,^15,31,39^ as there was no way of knowing if the teeth had been lost to periodontitis or an early dentition changeover of deciduous teeth.

**Figure 2.**
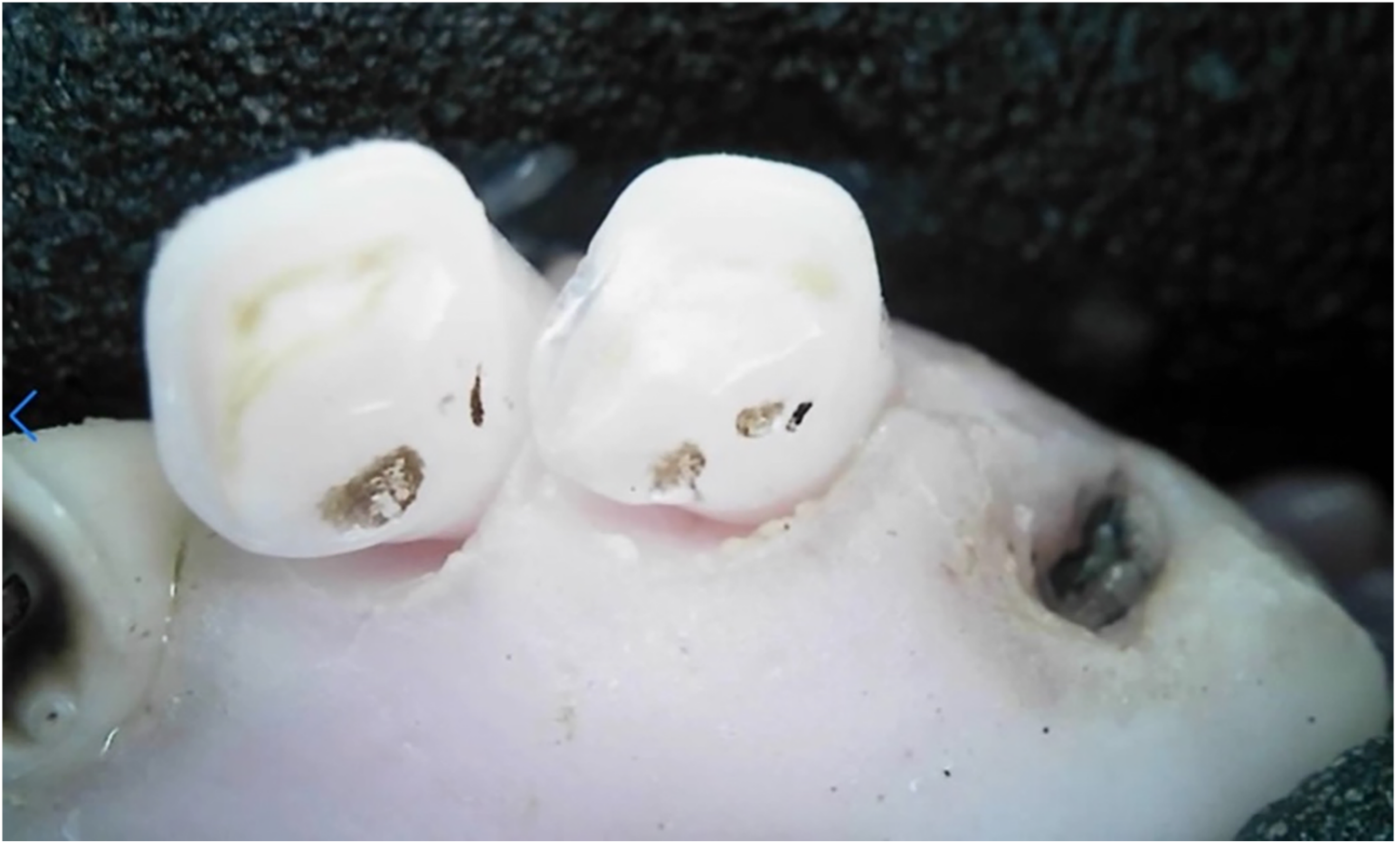
Retained root remnants of the deciduous right canine tooth (804).

Two sheep were observed with retained root remnants, classified as Type II. Type II root remnants result from the spontaneous fracture of the dental crown, often due to caries or other factors, and remain exposed to the oral cavity and fluids.^40^ Such remnants may cause pain, inflammation, or pathological changes.^41^ The presence of root remnants was documented as incisor loss (Figure 2).

**Figure 3.**
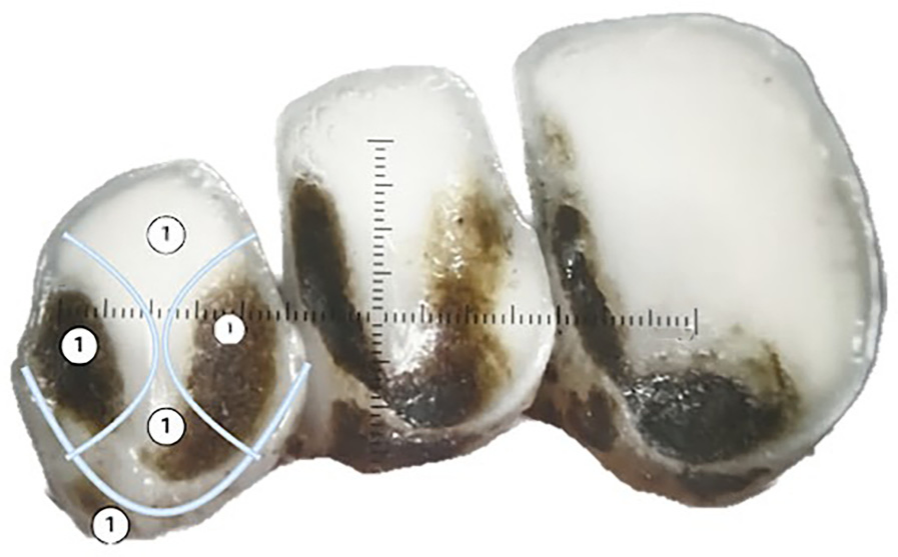
Adapted Rustogi Modified Navy Plaque Index^42^ used to assess the lingual side of sheep incisors for plaque. Note a score of 1 is assigned to each area with visible plaque.

**Figure 4.**
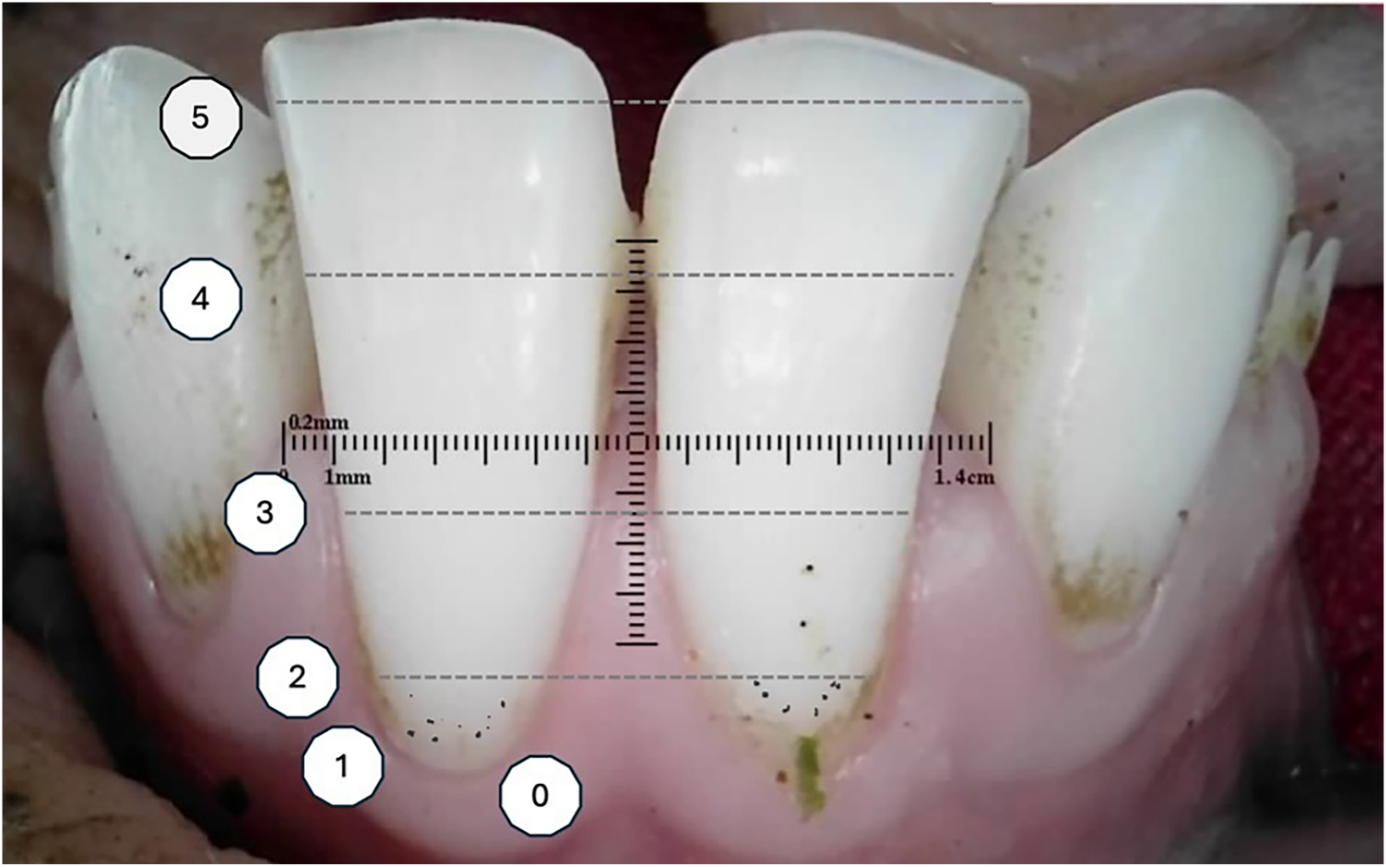
Turesky modification of the Quigley-Hein plaque index (TQHPI)^43^: 0—no plaque; 1—separate flecks of dental plaque at the cervical margin; 2—a thin continuous band of plaque (up to 1 mm) at the cervical margin; 3—a band of plaque wider than 1 mm but covering less than one-third of the crown; 4—plaque covering at least one-third but less than two-thirds of the crown; and 5—plaque covering two-thirds or more of the crown.

With no existing plaque assessment methods for sheep, 2 human plaque assessment techniques were adapted. Plaque was assessed on incisors’ lingual and buccal aspects, each tailored for a maximum score of 5. An adapted Rustogi Modified Navy Plaque Index^42^ was utilized to score the lingual side of the incisors, where a score of 1 was assigned to each area with visible plaque, and the total score was recorded (Figure 3). The buccal surface was assessed using the Turesky modification of the Quigley-Hein plaque index^43^ (Figure 4).

**Figure 5.**
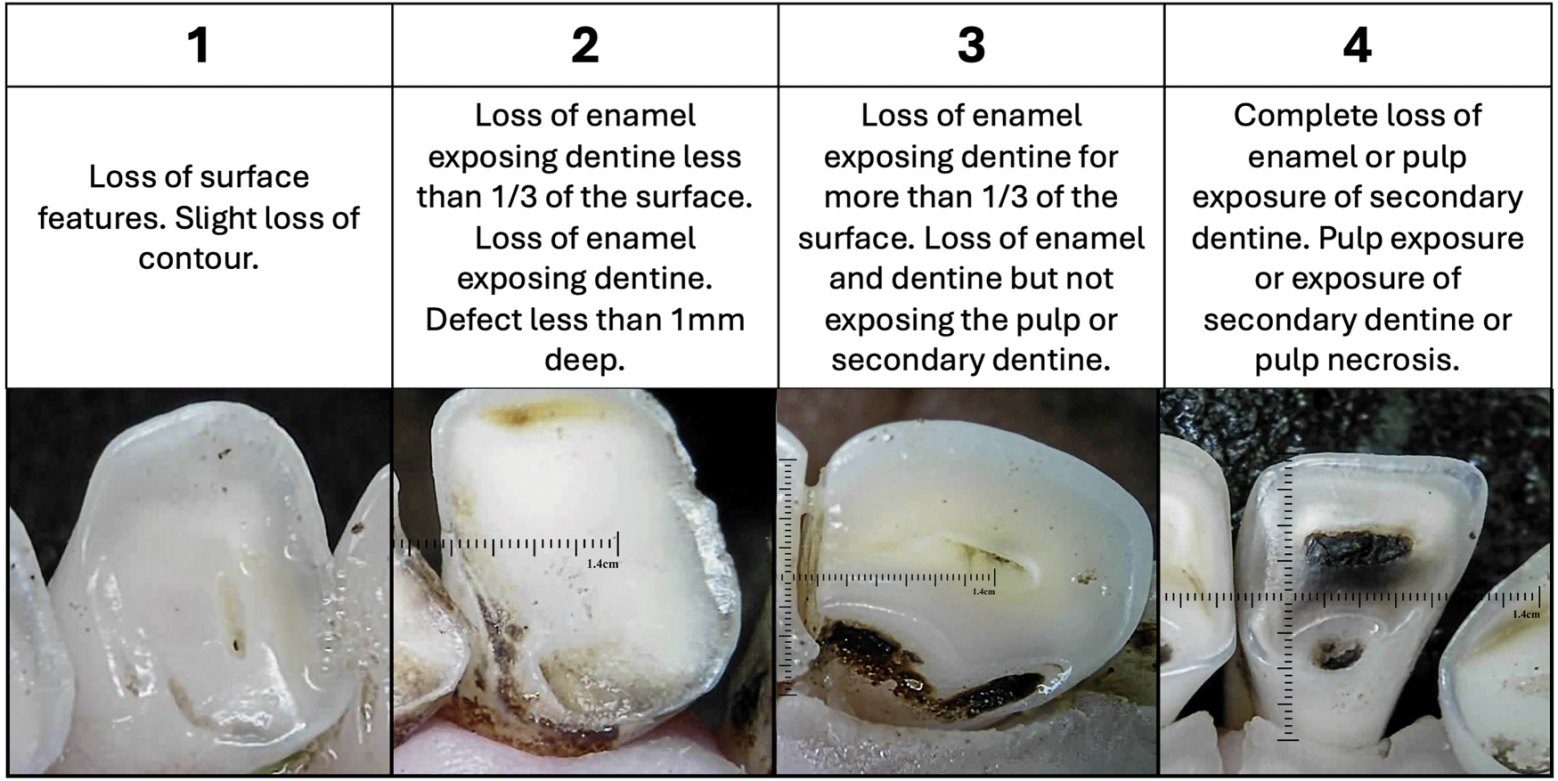
Qualification criterion to detect pathology of tooth wear—grades 1 to 4.^45^

This study used the photographic diagnostic method for caries assessment.^44^ Despite certain limitations, this approach provided an acceptable level of diagnostic accuracy for detecting caries. Incisors were classified as either sound or carious, with visible lesions, including active initial and arrested caries, categorized as carious. The Smith and Knight Tooth Wear Index assessed wear on the incisors.^45^ Each incisor was given a single Tooth Wear Score from 1 to 4 (Figure 5).

## Results

### Missing Incisors

In the 10-month-old sheep group, the deciduous right first incisor (801) was the most frequently missing tooth, which accounted for 45% of all missing teeth in this age group. Other notable missing teeth positions included the deciduous left first incisor (701) (18%) and the deciduous left third incisor (703) (14%), while the remaining positions had lower percentages—deciduous right canine (804) (9%), deciduous right third incisor (803) (5%), deciduous right second incisor (802) (5%), deciduous left canine (704) (5%), and the deciduous left second incisor (702) (0%).

In the 20-month-old sheep group, the most significant tooth loss was observed with tooth 404 (24%), 403 (22%), and 304 (16%). Tooth 303 showed the highest percentage of missing teeth in this age group, with 29%, while no missing teeth were observed in teeth 301 and 401.

### Plaque

**Figure 6.**
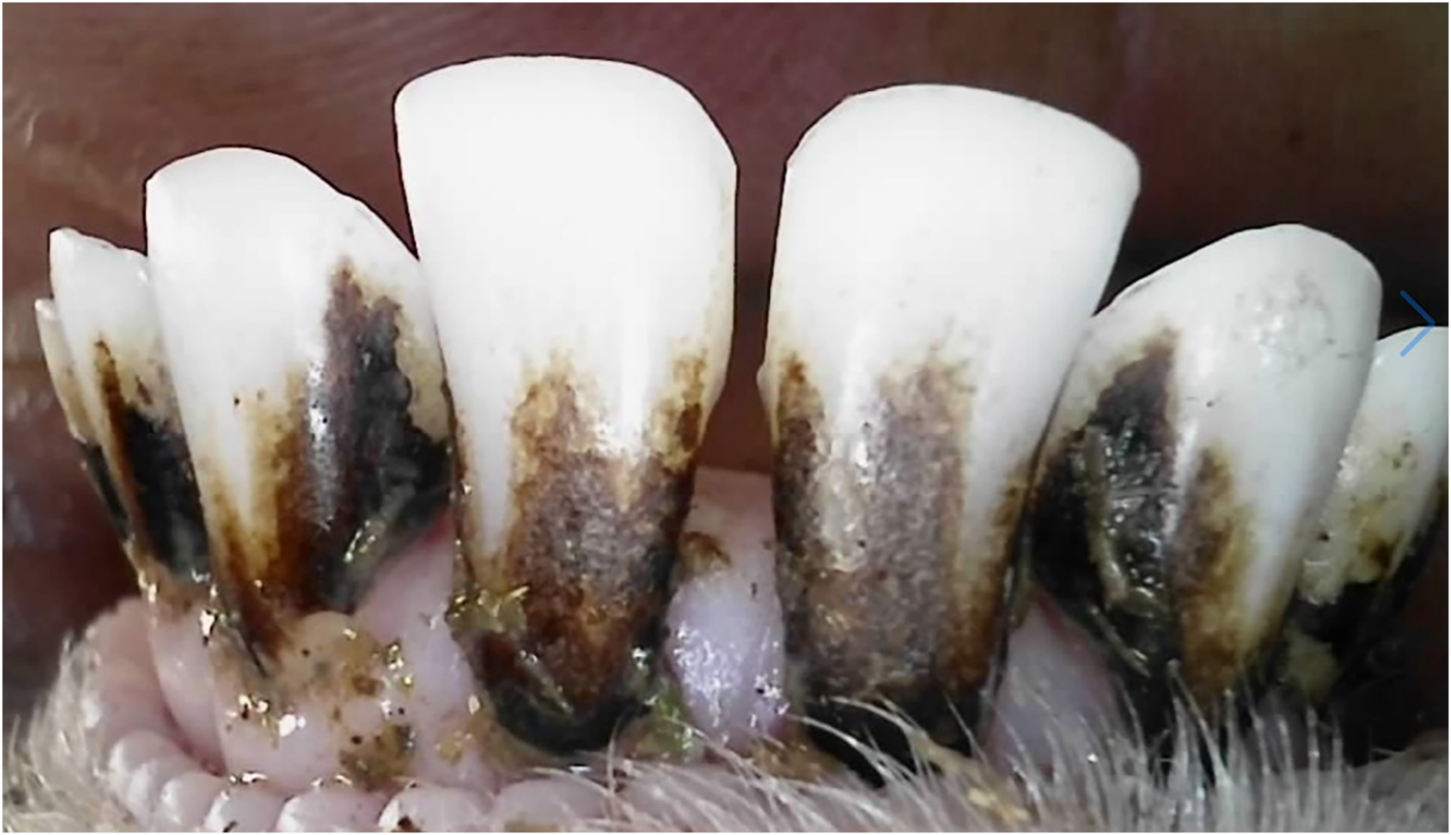
A 10-month-old ewe with heavy plaque covering over a third of the tooth surface.

The plaque scores for both 10-month and 20-month sheep showed a range of plaque accumulation across different surfaces. Figure 6 shows a 10-month-old ewe with heavy plaque covering over a third of the tooth surface.

**Figure 7.**
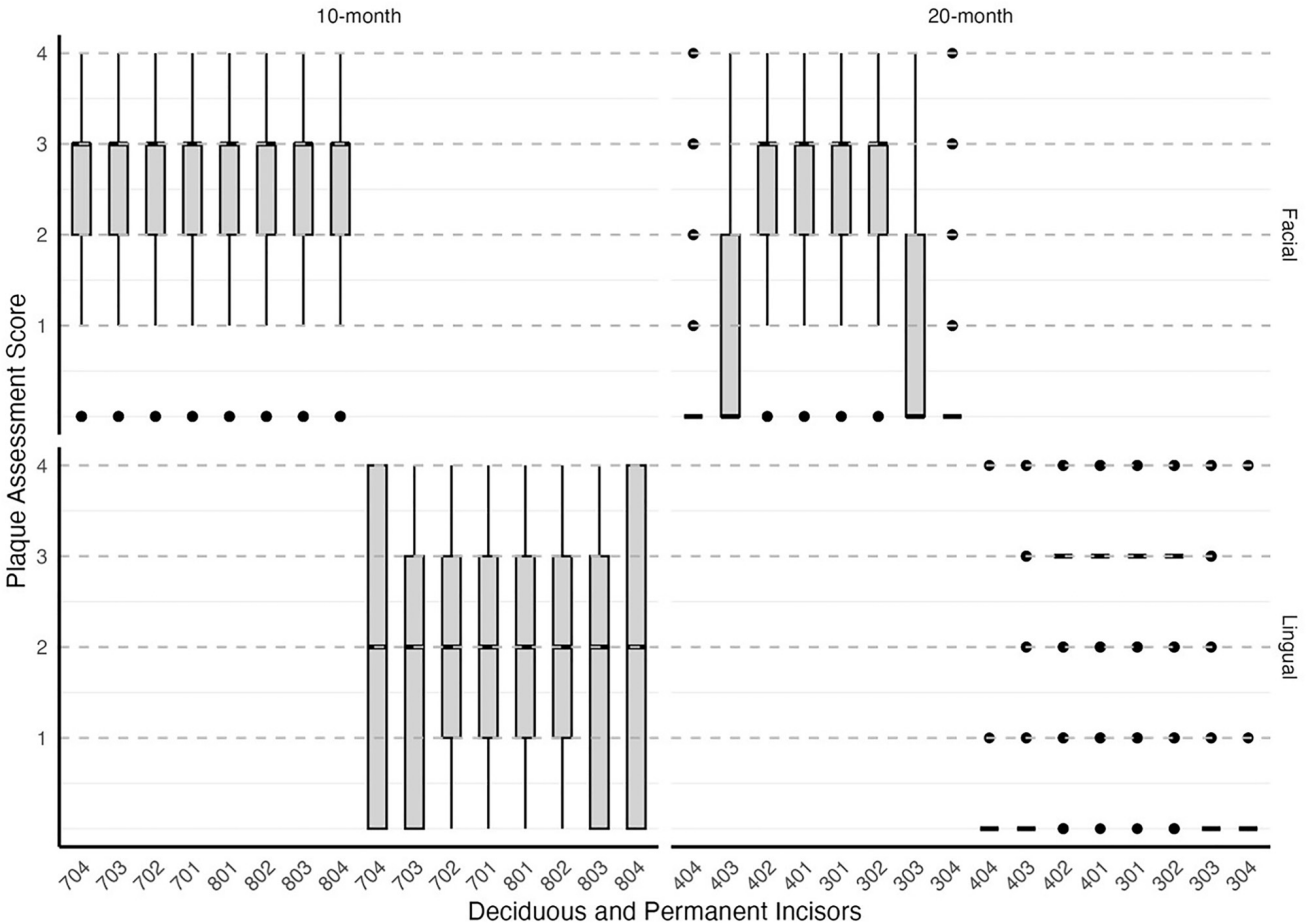
Distribution of plaque scores across deciduous and permanent incisors by age group (10-month and 20-month) and surface (buccal and lingual). The boxes represent the interquartile range (IQR) of plaque scores, indicating the middle 50% of observed scores for each tooth. The line within each box denotes the median score. Whiskers extend to the minimum and maximum scores within 1.5 times the IQR from the first and third quartiles, reflecting the overall data spread. Outliers beyond this range are not present in the dataset. Plaque accumulation was higher on lingual surfaces in the 10-month group and buccal surfaces in the 20-month group. Teeth are labeled using the Modified Triadan System, with deciduous incisors (704-804) for 10-month-old sheep and permanent incisors (304-404) for 20-month-old sheep.

In the 10-month age group, the median plaque score was higher on the buccal surface, median of 3 ± IQR 1, than on the lingual surface, median of 2 ± IQR 2; in the 20-month group, the plaque scores were each median of 2 ± IQR 3, on buccal and lingual surfaces. Both age groups showed the same maximum plaque score of 4 and a minimum of 0 across all surfaces, indicating variability within each group. The distribution of plaque scores across the 10-month and 20-month ewes (Figure 7) reflects variability in plaque accumulation by tooth and surface type. The 10-month-old sheep exhibit generally higher plaque scores on the buccal surface, with a concentration around scores of 2 and 3, while the 20-month-old sheep show a wider range of buccal plaque scores. The distribution of lingual plaque scores in 20-month-old sheep exhibits a limited range across the assessed teeth, with most teeth scoring consistently low or zero. This restricted variability resulted in the absence of traditional box and whisker structures for several teeth, as there was insufficient spread in the data to generate quartiles. Teeth 401-404 had consistently low scores, with a median of 0.

### Caries

**Figure 8.**
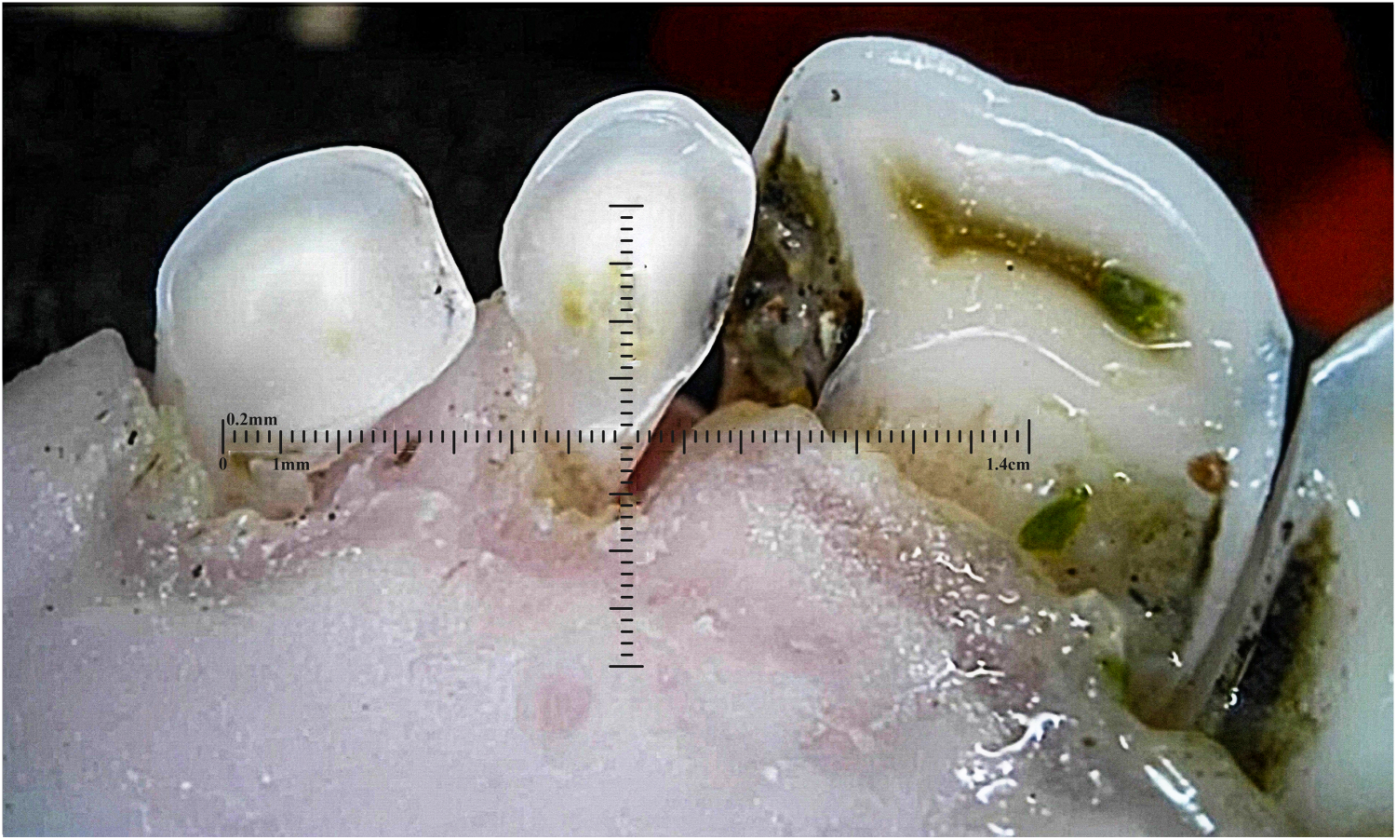
Dental caries on permanent left third incisor (303).

**Figure 9.**
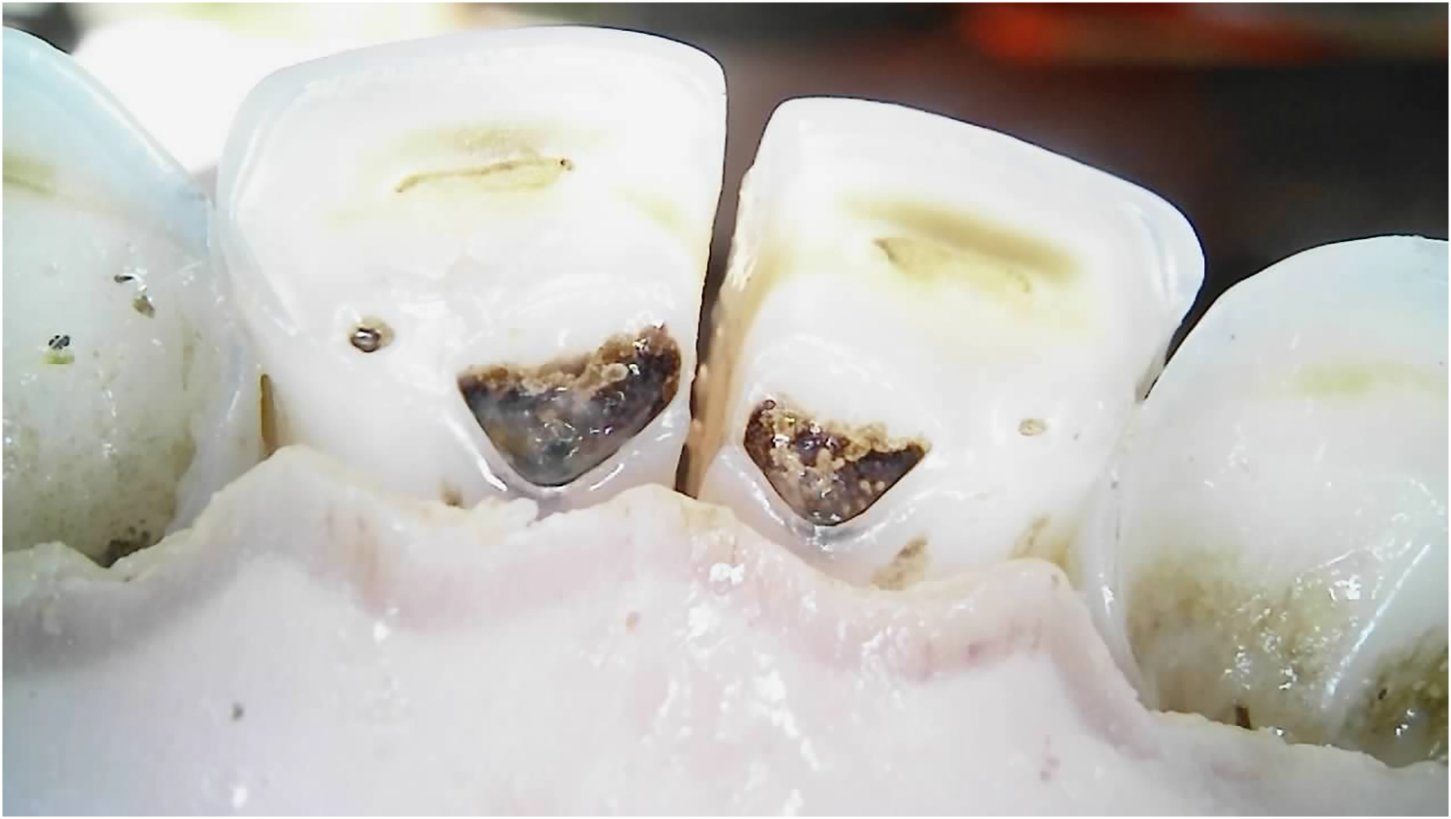
Dental caries in fissures on permanent right (401) and left (301) first incisor teeth.

Dental caries was assessed solely within the permanent incisors of the 20-month-old sheep, with a prevalence of 5% exhibiting caries in one or more incisors. The carious lesions predominantly emerged in the wear patterns within the pits and fissures (Figures 8 and 9).

### Wear

**Figure 10.**
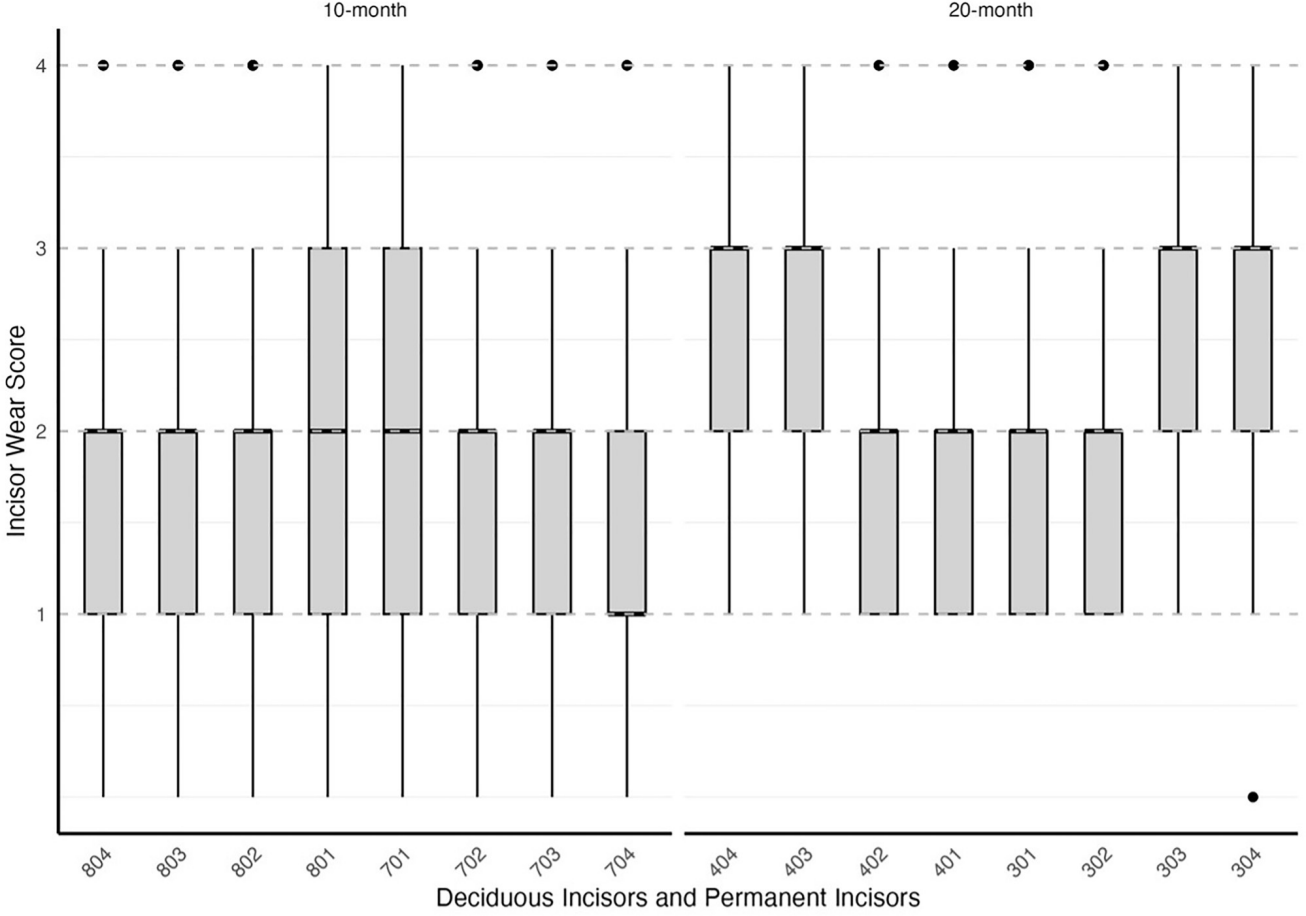
Distribution of tooth wear scores across deciduous and permanent incisors by age group (10-month and 20-month). The boxes represent the interquartile range (IQR) of tooth wear scores, indicating the middle 50% of observed scores for each tooth. The horizontal line in the box represents the median. Whiskers extend to the minimum and maximum scores within 1.5 times the IQR from the first and third quartiles, reflecting the overall wear range. Outliers beyond this range are not present in the dataset. Tooth wear was more prominent in the 20-month group, particularly on permanent incisors, indicating age-related wear progression. Teeth are labeled using the Modified Triadan System, with deciduous incisors (804-704) for 10-month-old sheep and permanent incisors (404-304) for 20-month-old sheep.

The tooth wear scores for both 10-month and 20-month sheep ranged from 0 to 4, with a median of 2 ± IQR 1 in both age groups, indicating similar variability in tooth wear scores across the ages. Figure 10 illustrates that wear is distributed across all incisors, with some higher scores (outliers) observed, particularly in the 10-month group.

**Figure 11.**
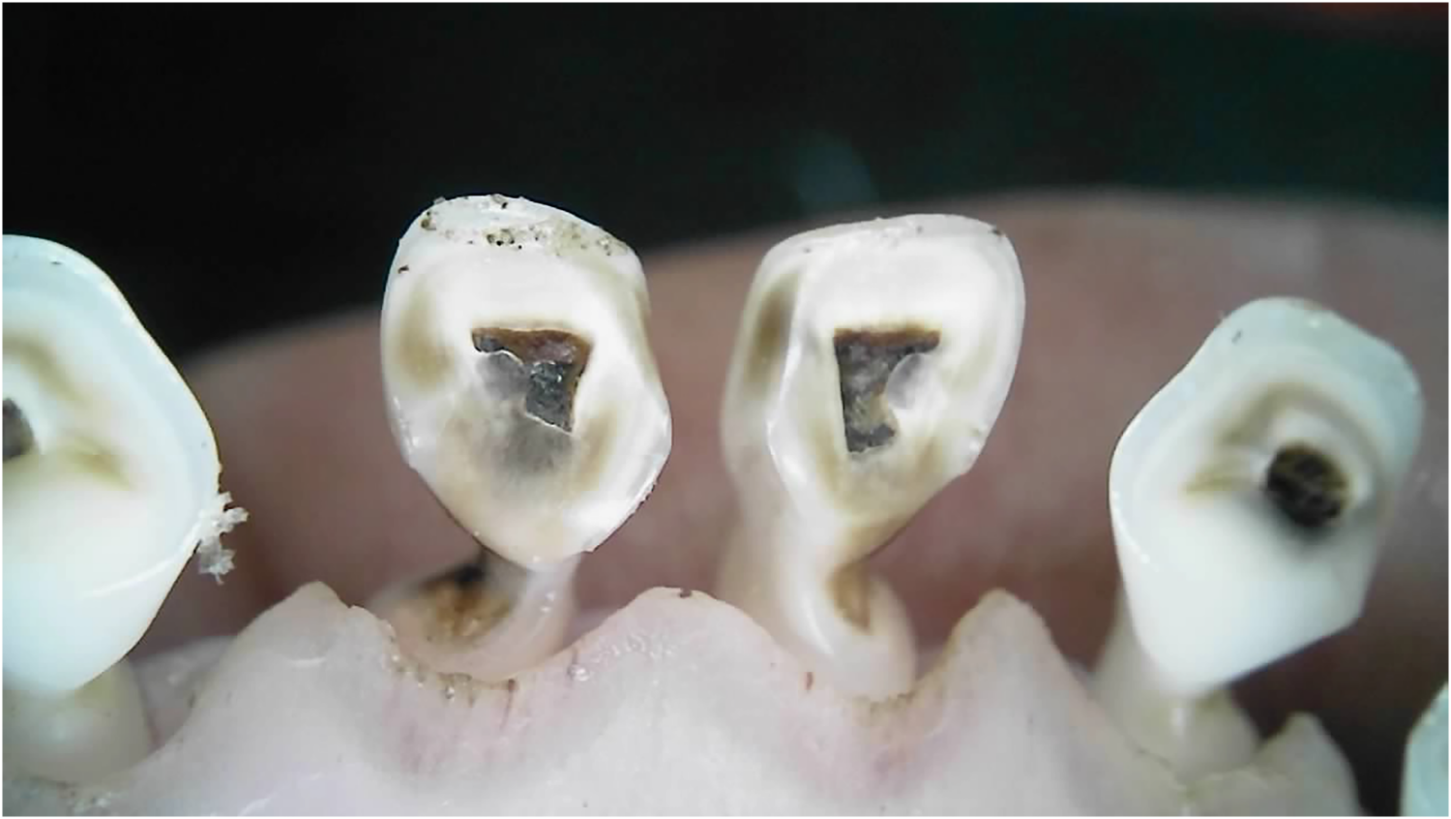
Wear of deciduous right (801) and left (701) first incisor teeth with pulp necrosis in a 10-month-old ewe.

**Figure 12.**
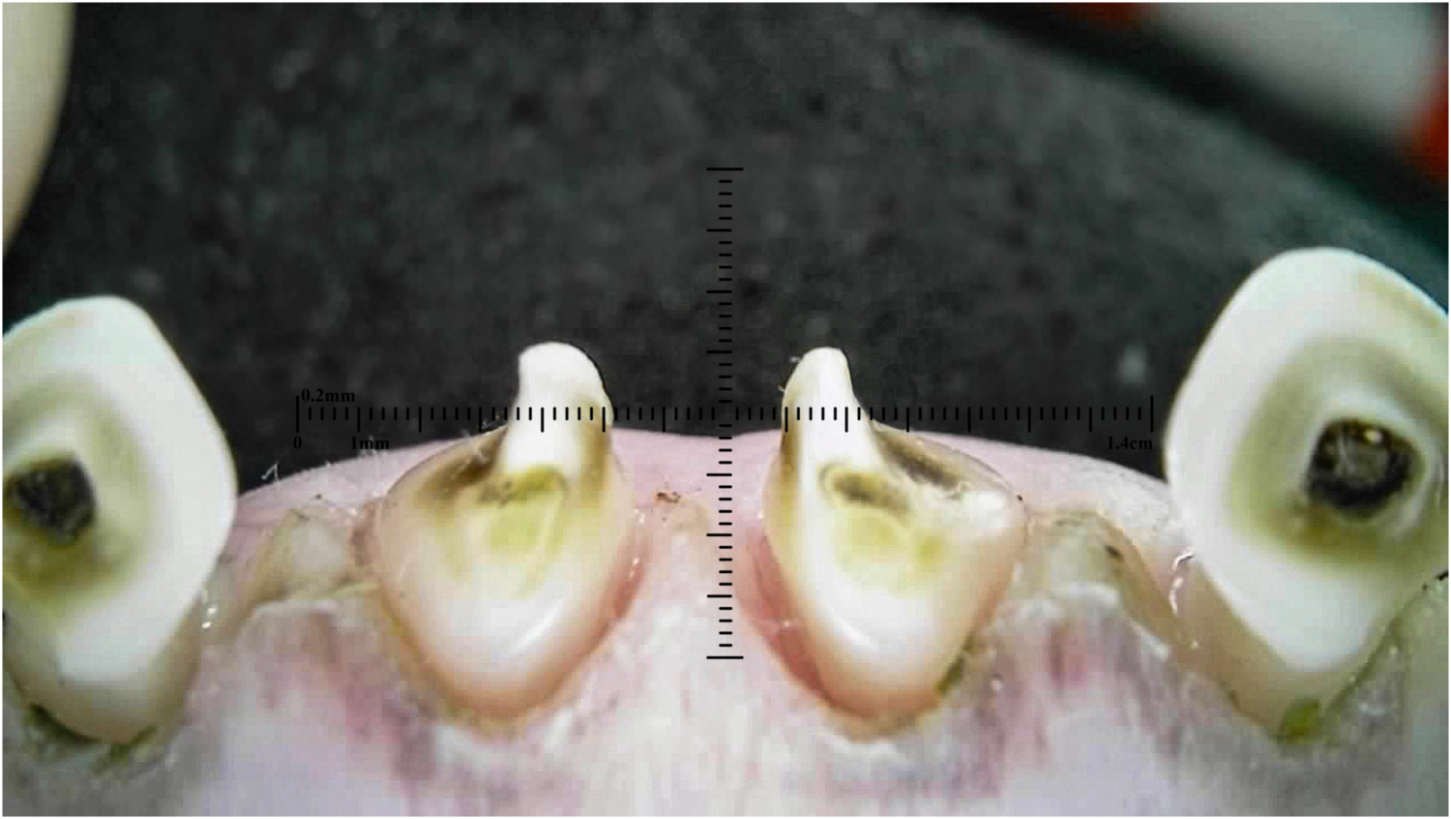
Incisor remnants on the deciduous right (801) and left (701) first incisor teeth in a 10-month-old ewe.

**Figure 13.**
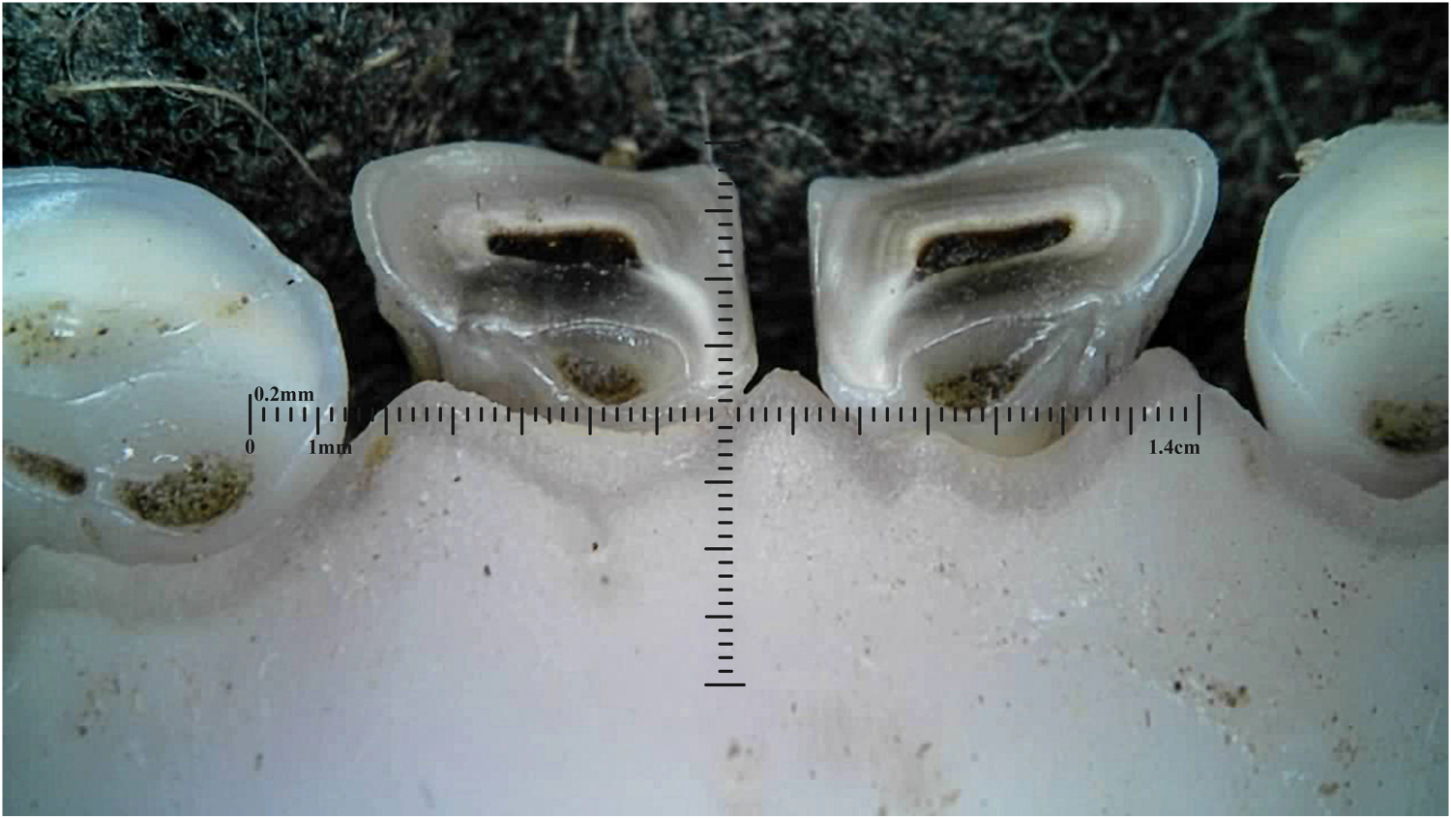
Wear, pulp necrosis, and crown discoloration on the deciduous right (801) and left (701) first incisor teeth in a 10-month-old ewe.

**Figure 14.**
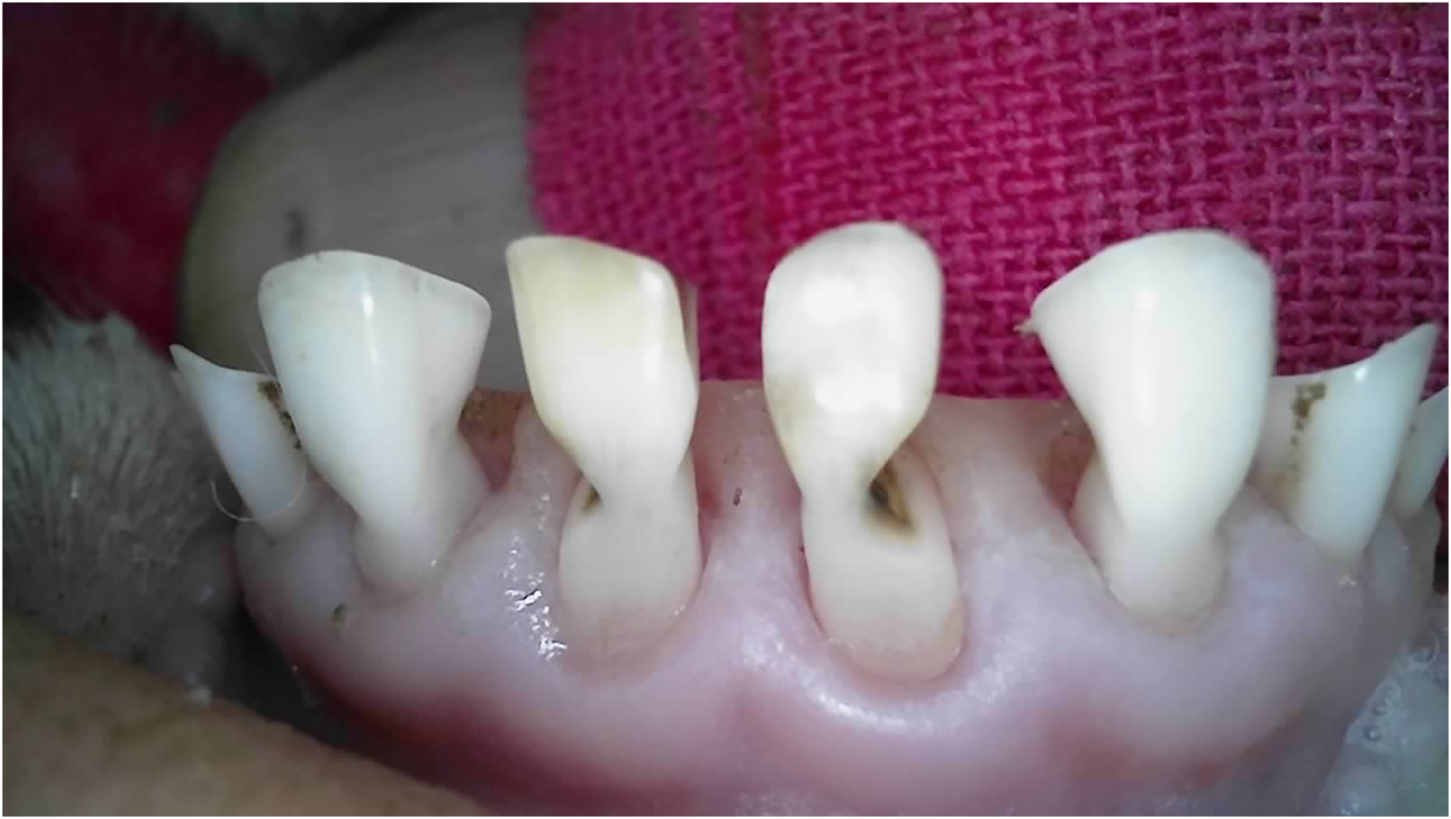
Wear of the deciduous right (801) and left (701) first incisor teeth in a 10-month-old ewe.

**Figure 15.**
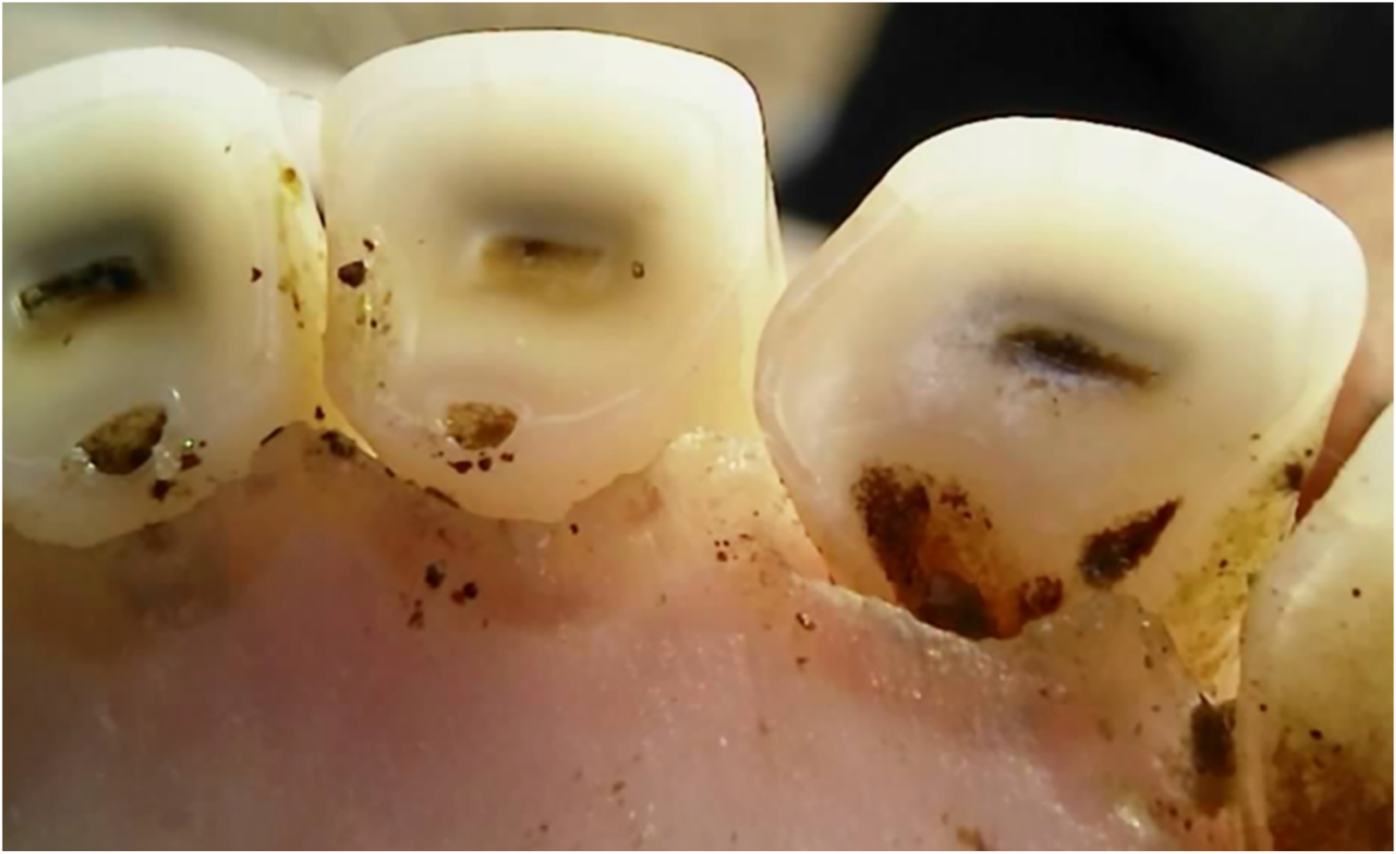
Wear and pulp necrosis of the permanent right first (401) and second (402) and left first (301) incisor teeth in a 20-month-old ewe.

Outliers can be seen in wear score 4, where 33% of the 10-month-old and 59% of the 20-month-old ewes were assessed as having at least one incisor with complete enamel or pulp exposure or secondary dentine loss. Figures 11 to 14 show pulp necrosis in 10-month-old ewes, and Figure 15 shows pulp necrosis in permanent incisors of a 20-month-old ewe.

### Twinning Anomalies

**Figure 16.**
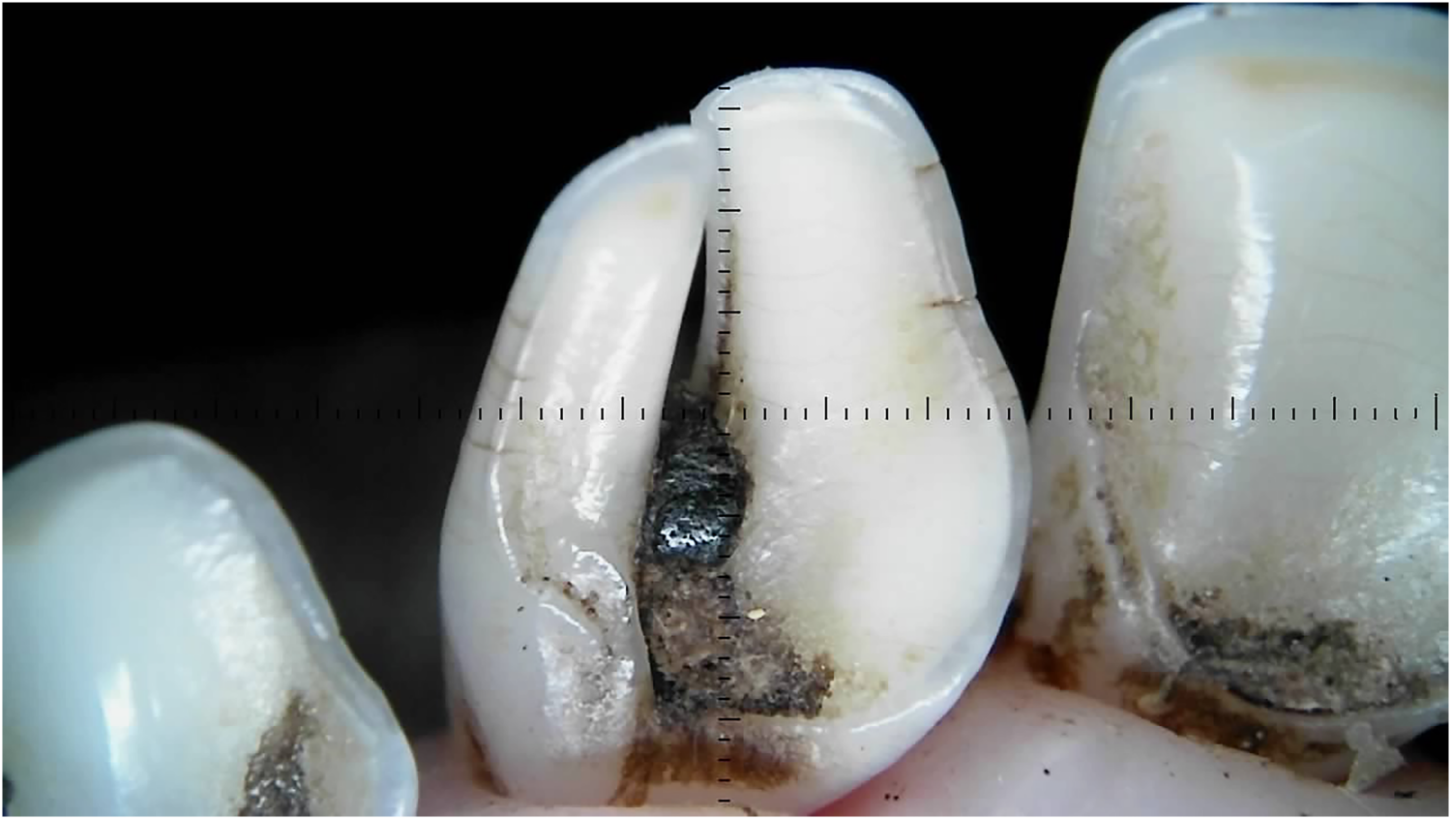
Gemination of deciduous right second incisor (802) in a 10-month-old ewe.

**Figure 17.**
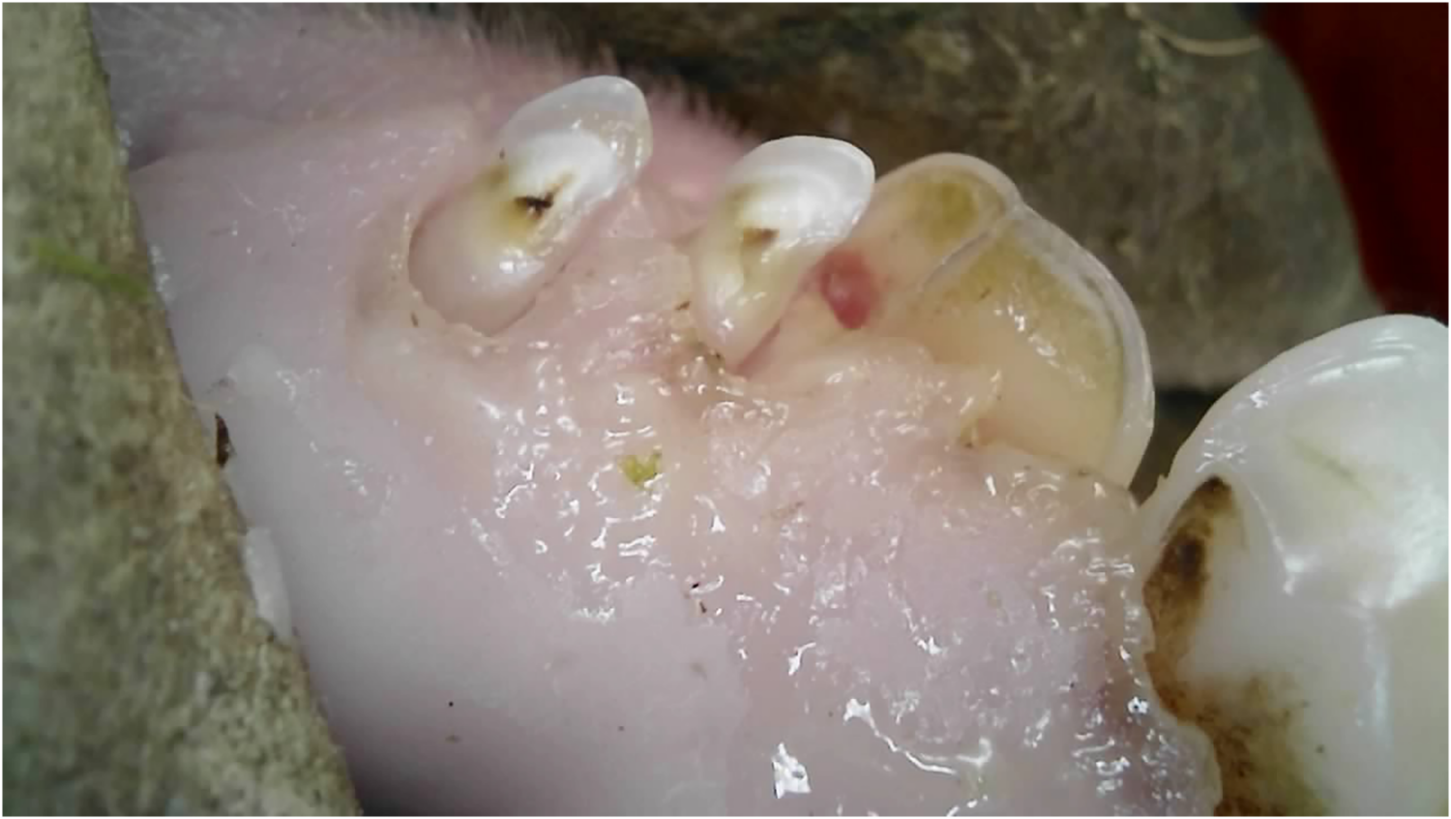
Gemination of a permanent left second incisor (302) in a 20-month-old ewe.

**Figure 18.**
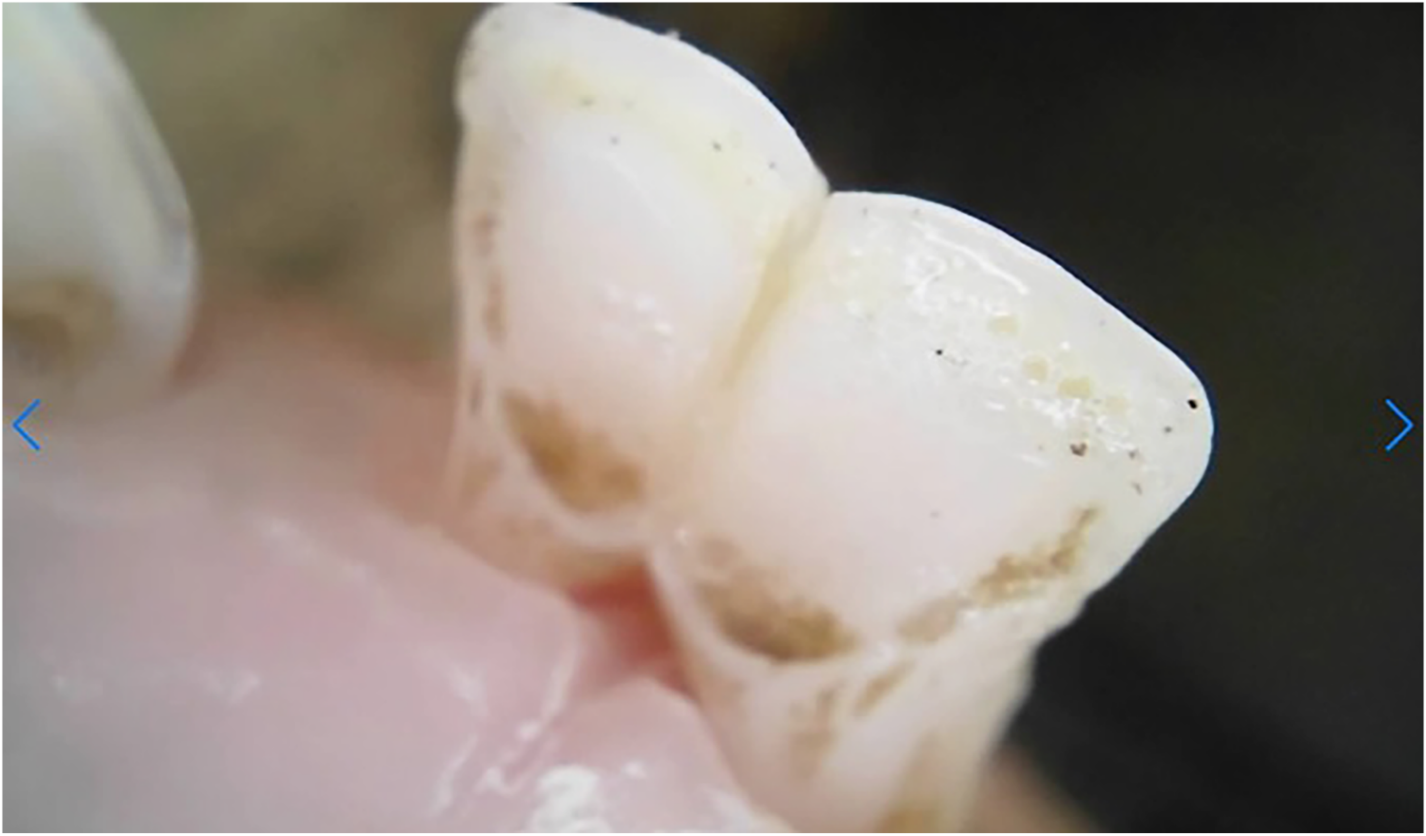
Fusion of the deciduous right (801) and left (701) first incisor teeth in a 10-month-old ewe.

Two cases of gemination were identified. One geminated incisor was observed in a 10-month-old ewe (Figure 16), and the other in a 20-month-old ewe (Figure 17). One sheep was assessed with fused incisors on the deciduous first incisors (Figure 18).

### Talon Cusp

**Figure 19.**
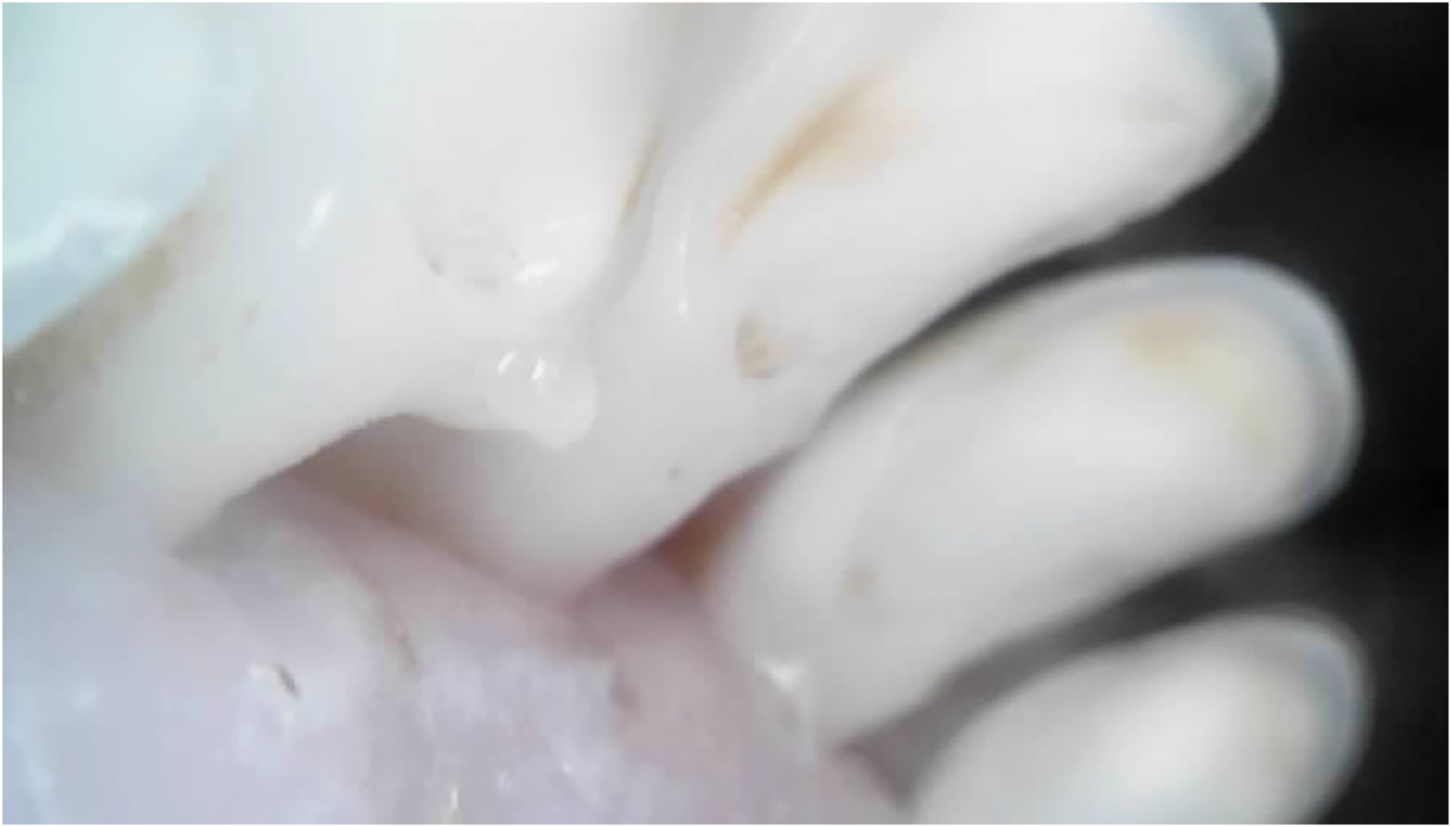
Talon cusp on deciduous left first incisor tooth (701) in a 20-month-old ewe.

One sheep was assessed with a talon cusp on a deciduous first incisor (Figure 19).

### Localized Enamel Hypoplasia

**Figure 20.**
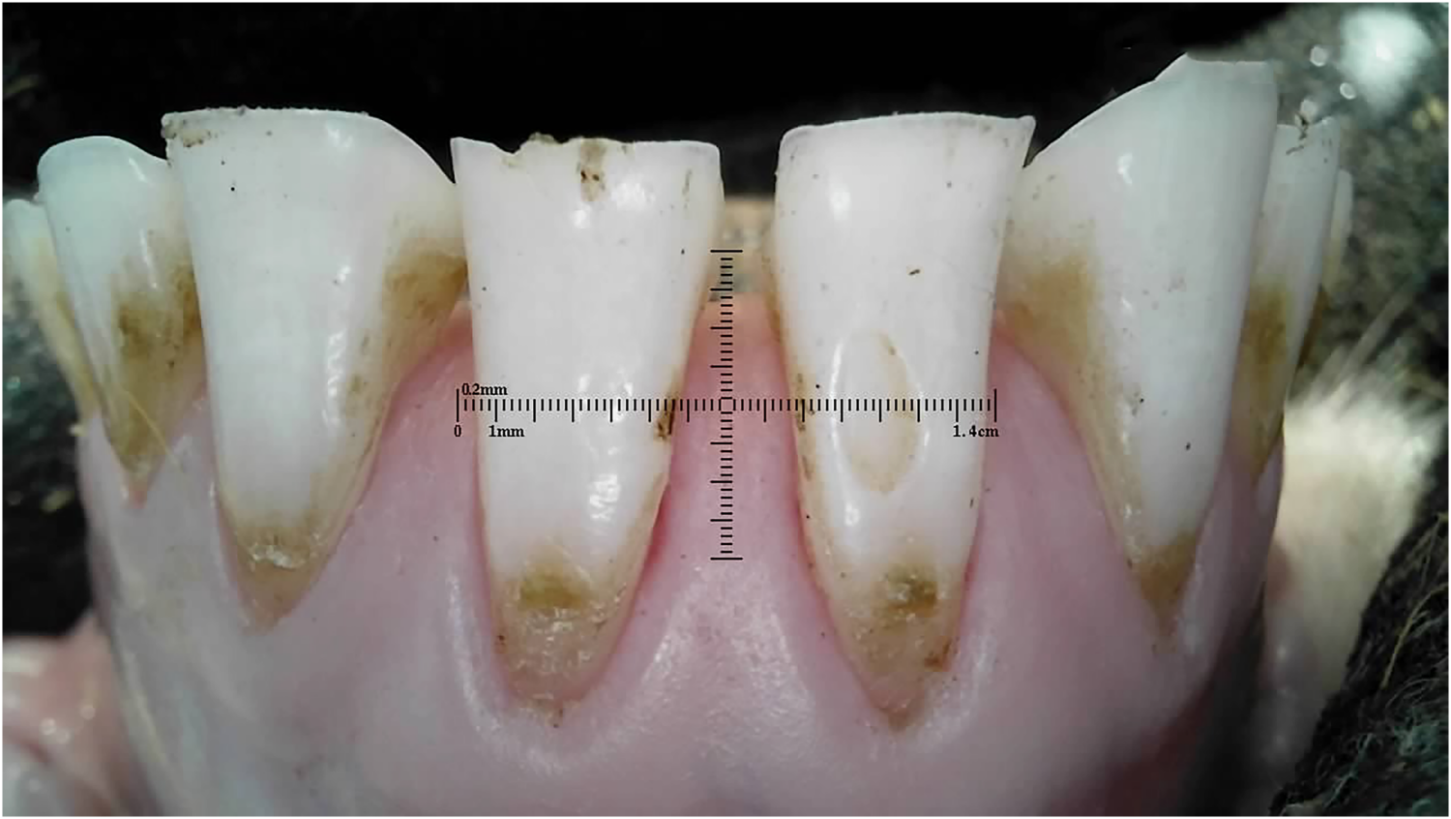
Localized hypoplasia on deciduous right (801) and left (701) first incisor teeth in a 10-month-old ewe.

**Figure 21.**
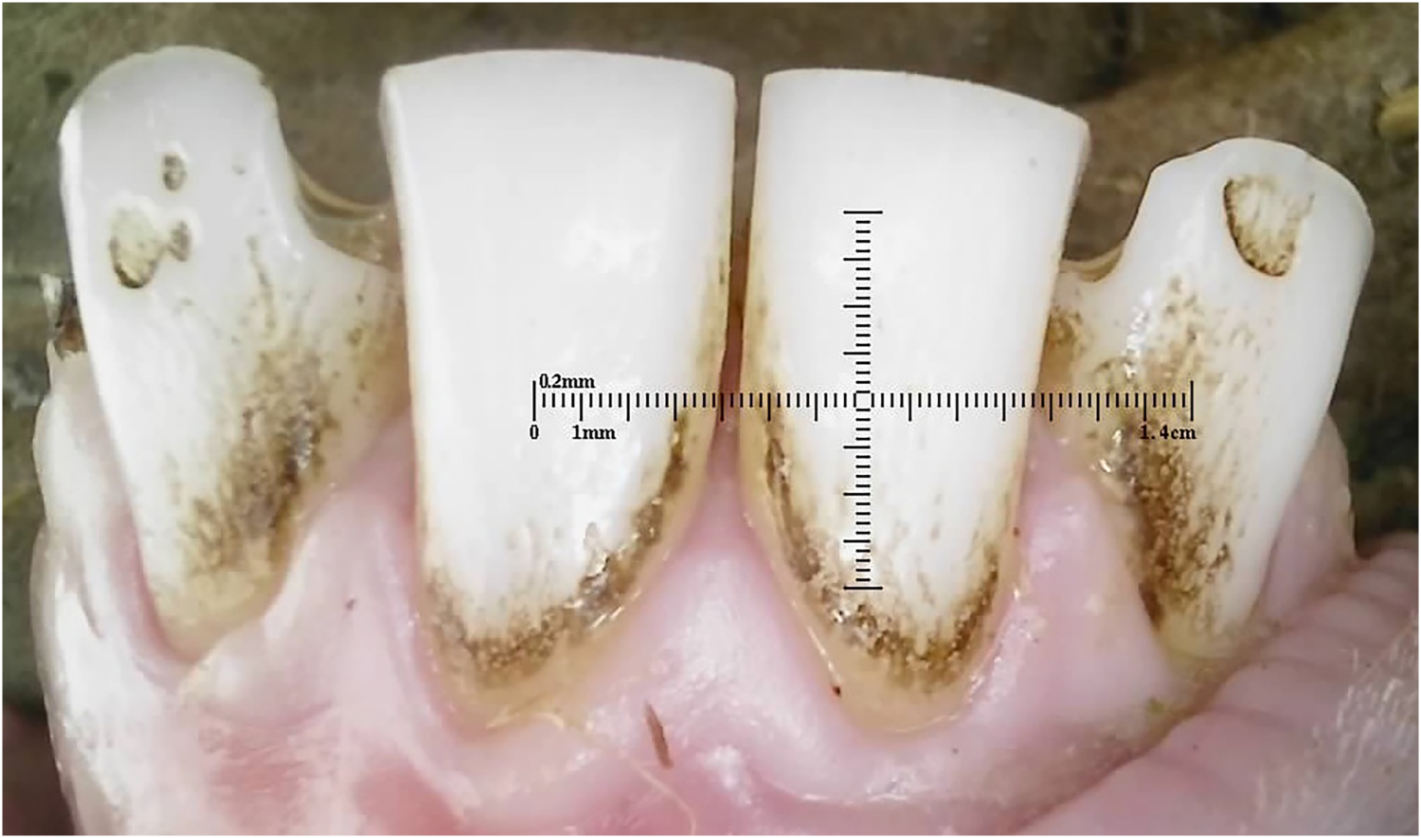
Localized hypoplasia on permanent right (402) and left (302) second incisor teeth in a 20-month-old ewe.

Localized enamel hypoplasia in both groups predominantly appeared as shallow pits measuring 2 to 3 mm in diameter, shown in the 10-month-old (Figure 20) and the 20-month-old (Figure 21). In the current study, localized enamel hypoplasia was observed in the 10-month-old and 20-month-old sheep, with an incidence of 1% in each group. In both age groups, teeth 701, 801, 301 and 401 were the most affected teeth with localized enamel hypoplasia.

### Amelogenesis Imperfecta

**Figure 22.**
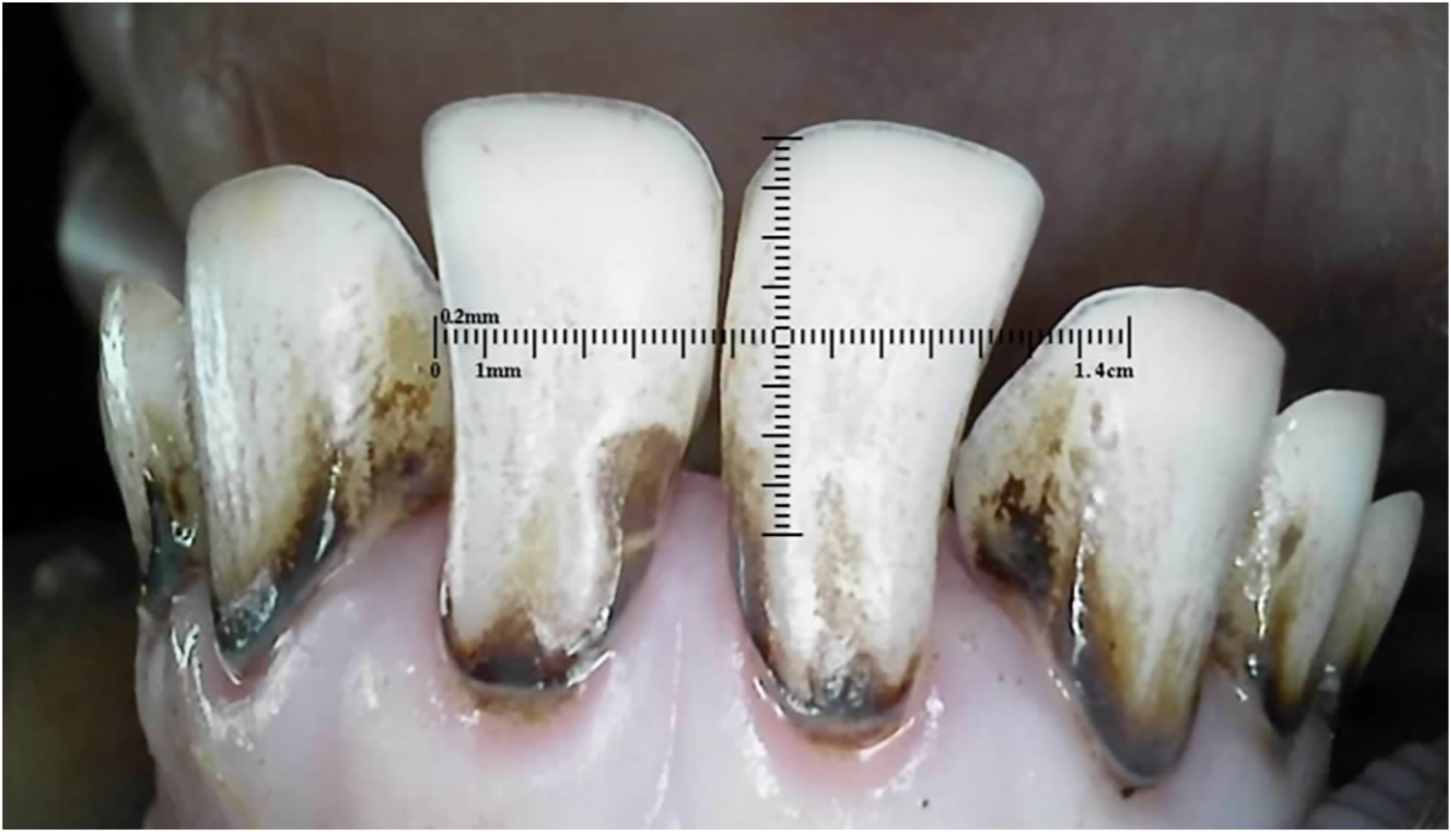
Deciduous incisor teeth with suspected Amelogenesis imperfecta in a 10-month-old ewe.

**Figure 23.**
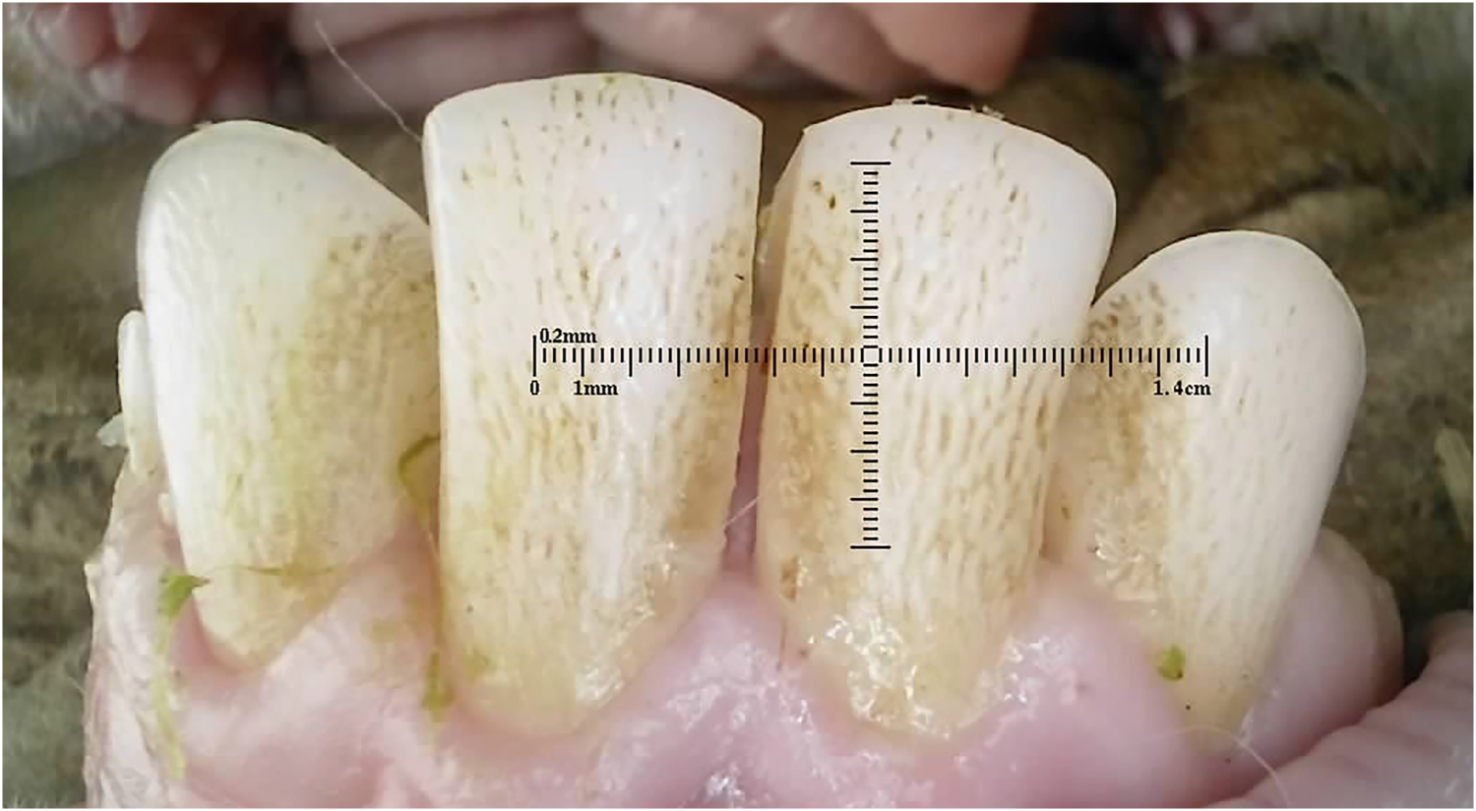
Permanent incisor teeth with suspected Amelogenesis imperfecta in a 20-month-old ewe.

**Figure 24.**
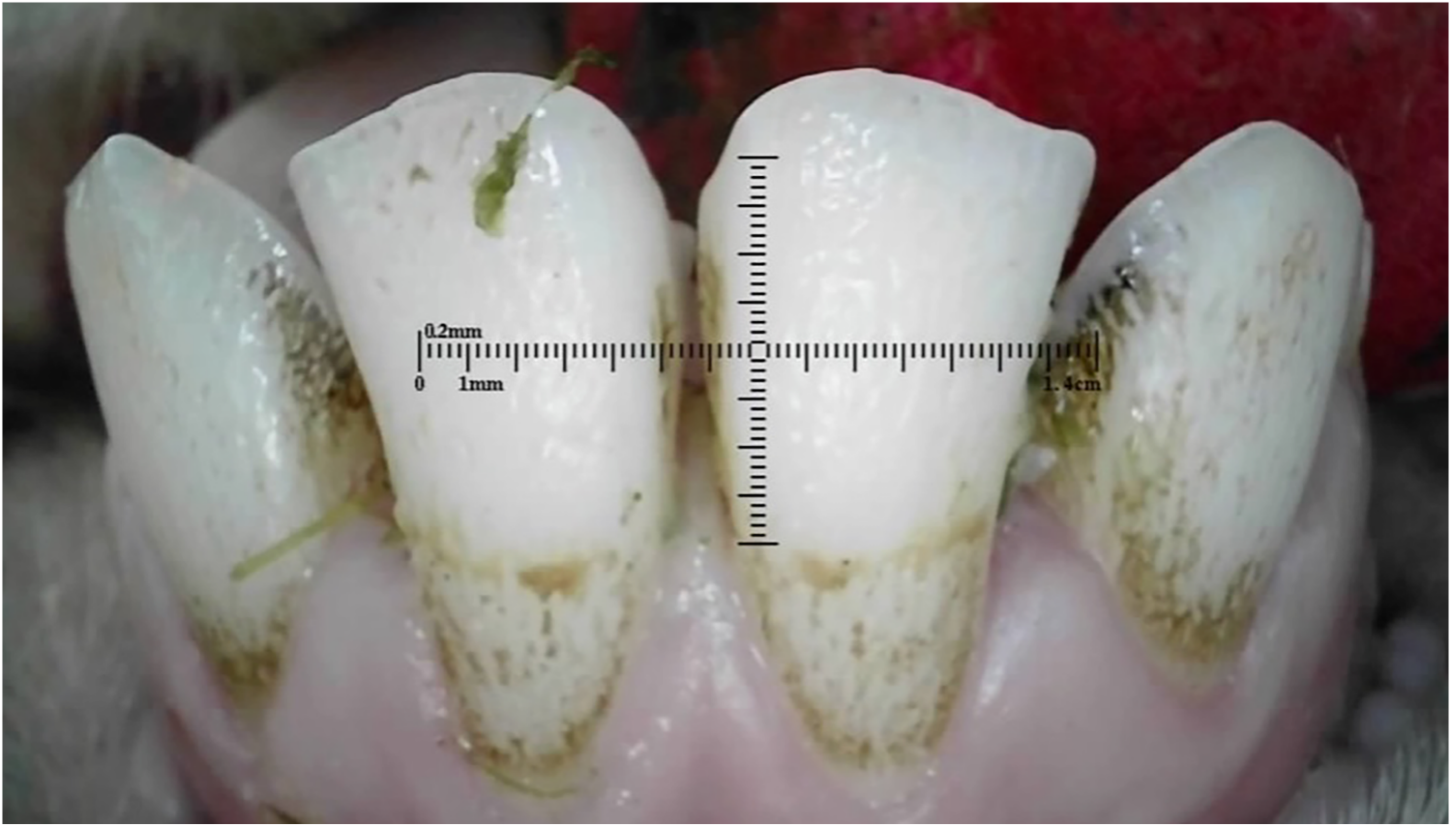
Permanent incisor teeth with suspected Amelogenesis imperfecta in a 20-month-old ewe.

The presentation of suspected Amelogenesis imperfecta in both age groups involved mottling and pitting of all incisors, similar to those previously documented in sheep with excessive wear in New Zealand.^33^ Amelogenesis imperfecta was seen in 19 (1%) of 10-month-old Merino ewes (Figure 22) and 159 (37%) of 20-month-old Dohne Merino ewes (Figures 23 and 24).

## Discussion

This study represents the first comprehensive investigation into the prevalence and diversity of dental anomalies affecting incisor health in sheep, identifying a range of conditions previously undocumented in the species. Notably, conditions such as gemination, talon cusp formation, and Amelogenesis imperfecta were observed, highlighting the incisor pathologies in sheep. The findings reveal patterns in tooth wear, plaque accumulation, and incisor loss across age groups, underscoring age and environmental factors as key influences.

### Incisor Loss

The analysis of missing teeth by tooth position reveals distinct patterns between the 10-month-old and 20-month-old sheep. In the younger group, tooth loss was primarily concentrated in the central incisor position, accounting for nearly half of all missing teeth in the 10-month-old group. This position represents a central incisor, which is expected to be shed and replaced as part of the natural dental transition occurring around 12 months of age. Therefore, it is expected to observe missing incisors in this position in yearling sheep. However, without observing the missing tooth, it is difficult to determine the specific cause of the incisor loss, as the lack of an emerging incisor led to it being recorded as missing. In the older group, tooth loss was more evenly distributed across multiple positions, with teeth 304, 403, and 404 showing significant tooth loss. The highest percentage of missing teeth was 303. The more widespread pattern of missing teeth in 20-month-old sheep may indicate a progression of wear and dental health issues with age, particularly for permanent incisors, as this group showed more uniform loss across several positions compared to the younger sheep.

The differences in tooth loss between age groups highlight the importance of monitoring specific tooth positions for early signs of dental wear or disease, as the progression of tooth loss seems to increase with age, affecting a broader range of teeth. The 10-month-old age group had missing 804 and 704 teeth, being 9% and 5%, respectively, while the 20-month-old group had missing incisors 404 and 304, being 24% and 16%, respectively. Eruption of teeth 404 and 304 can occur at approximately 4 years of age; however, this can vary by as many as 8 months. These teeth show the most variable eruption times of all incisors and are unerupted in up to 8% of sheep.^16,18,46^ Sheep deciduous incisor teeth 804 and 704 have been found to have the lowest mean total volume compared to central incisors and the lowest resistance to compression test of all incisors.^47^ The lack of volume and resistance to compression may be contributing factors in the observations in this study of the prevalence of loss of 404 and 304.

It is recognized that there are limitations with assessments of photographs that may have restricted the ability to identify emerging incisors. However, several of these missing incisors had pulp necrosis on neighboring teeth, but without observing the lost tooth, it would be hard to judge other than to say it had been lost prematurely. All incisors deemed missing were assessed as deciduous and identified as outside the usual dentition pattern. This indicated a premature disruption in the natural dentition changeover, with many absent teeth occupying positions deemed early for this transition.

### Plaque

Dental plaque is the precursor to dental calculus, a hard deposit formed through calcification. Primarily composed of calcium phosphate, calculus forms both supra- and subgingival and plays a significant role in the development and progression of periodontal disease.^48^ While much of the research into plaque and calculus focuses on human health due to the association with caries and periodontal disease, the understanding of these conditions in animals, notably sheep, remains limited.^48^ The findings highlight that tooth surface influences plaque accumulation in sheep incisors, with distinct patterns observed between the 10-month and 20-month age groups. The 10-month-old various environmental conditions and genetic backgrounds may have introduced additional variability in plaque scores. The 20-month-old sheep were sourced from a single flock on one farm, providing a more uniform environment but limiting the broader applicability of their results. The lack of box and whiskers for certain teeth in the 20-month-old sheep lingual plaque scores indicates a minimal range in plaque accumulation. The consistently low scores suggest that lingual surfaces in this age group either have inherently lower plaque retention or that other factors at this age minimize plaque development. The consistency in low scores across teeth provides valuable insight into plaque deposition dynamics and could inform management strategies to maintain oral health in aging sheep. It may point to age-related changes in plaque accumulation patterns; however, with the 10-month difference, there was only a little difference to make age-related assumptions at this stage without further research.

### Dental Caries

In this study, dental caries was observed on the permanent incisors of 5% of the 20-month-old sheep. Carious lesions predominantly formed within pits and fissures of the teeth, consistent with findings observed in humans.^49^ Dental caries has historically been considered rare in sheep, with minimal research or documented cases. One of the few discussions of sheep caries examines several affected sheep incisors, stating that caries occurs in younger sheep, with lesions forming around the necks of deciduous incisors that then snap off and leave a ragged stump.^15^ However, no published research explains how this consensus was reached, leaving significant gaps in the understanding of how caries progresses in sheep.

Two recent observational studies reported higher caries prevalence than seen in this study. In Iraq, caries was noted as the most common dental disorder in sheep, affecting 28% of the sample,^50^ while in Iran, 9% was reported.^51^ However, the diagnostic criteria used in these studies, including the specific location and methods for diagnosing caries, were not clearly defined, making it challenging to compare these findings with the current study or to assess caries’ prevalence in sheep populations fully. While pits and fissures are natural anatomical features of teeth, they can act as “trap” areas for food and bacteria, which increases the risk of caries development. In sheep, the lingual cingulid of the incisors creates a distinct lingual fossa.^47^ This unique morphology of sheep teeth suggests that these features may play a role in the susceptibility to caries. In humans, the systemic consumption of low doses of fluoride (e.g., through drinking water) is known to reduce the depth of pits and fissures during tooth development, decreasing their susceptibility to caries.^52^ It is yet to be discovered whether this fluoride effect also occurs in sheep. Only one site used fluoridated water as a water source, while the others were on rain-fed dams, streams, or bores.

The photographic approach to dental screening used in this study allowed for the effective detection of carious lesions, although this method has limitations. While photographic screening has been widely used in humans, particularly in younger children with primary dentition,^44^ its application in veterinary science, especially sheep, provides an innovative approach to diagnosing dental caries.

### Tooth Wear

The similarity in tooth wear scores between the 10-month and 20-month sheep suggests that age alone may not be a strong indicator of wear severity within these age groups. The consistent median and IQR values imply comparable levels of wear progression across these young and older ewes, potentially indicating that other factors, such as diet, environment, or genetics, may play a role, warranting further investigation.

### Twinning Anomalies

This study documented 2 twinning anomalies in sheep, with one instance of gemination observed in a deciduous incisor and one in a permanent incisor. These findings represent the first documented occurrences of gemination in sheep, as no previous reports exist in the literature. Twinning anomalies, which include gemination and fusion, are abnormalities in tooth shape. Gemination occurs when a single tooth germ partially divides, resulting in a large tooth with 2 distinct sections connected by a groove along with the incisal edge, while the total tooth count remains normal. This condition is characterized by a single crown that resembles a mirror image of 2 fused teeth, with a single root and canal.^22,53^ In contrast, fusion results from the union of 2 normally separate tooth germs, which can be complete or incomplete, depending on whether or not the teeth are joined along with the crown and root.^54^

In human dentistry, the aetiology of twinning anomalies is considered multifactorial, with genetic predisposition, environmental factors, and trauma proposed as potential contributing factors.^55,56^ These factors could similarly play a role in sheep, although the exact causes remain unknown. Functionally, these conditions can pose challenges, particularly if they disrupt normal tooth alignment or occlusion.^22^ While twinning anomalies in sheep are rare, further investigation could help determine any clinical significance these anomalies may have on sheep incisor function.

### Talon Cusp

Talon cusps are well-defined, morphologically altered cusp-like projections extending from the anterior teeth’ cingulum area and consist of enamel, dentine, and potentially pulp tissue.^57,58^ Although talon cusps have been extensively described in human dentistry, particularly in children and adolescents, their occurrence in sheep has not been previously reported. The exact aetiology of talon cusp formation remains uncertain, though genetic and environmental factors are thought to play a role in its development.^59^

### Localized Enamel Hypoplasia

Localized enamel hypoplasia occurs due to disruptions to the enamel organ during amelogenesis, specifically affecting only the developing teeth at the time and site of the insult rather than indicating a systemic issue.^60^ These defects are permanent, chronological stress indicators during enamel formation and are frequently linked to traumatic events, nutritional deficiencies, or inflammatory conditions.^61,62^ In sheep, enamel hypoplasia appears as shallow pits on newly erupted incisors.^31,33,63^ Studies from New Zealand in the 1970s and 1980s caused enamel hypoplasia to occur in sheep through experimental methods that damaged the developing tooth bud.^64^

In the current study, localized enamel hypoplasia was detected in both deciduous and permanent incisors. In the 10-month-old sheep, the lesions were confined to the central deciduous incisors, which suggests that the developmental insult occurred in utero, as amelogenesis of deciduous incisors in sheep starts around day 33 of gestation.^65^ In the 20-month-old sheep, the lesions on the permanent incisors were more pronounced, with some pits becoming confluent to form more significant defects. Since amelogenesis of the central permanent incisors begins around 5 to 8 months of age,^66^ the insult likely occurred during this developmental period. Localized enamel hypoplasia is a developmental abnormality, so the weakened or absent enamel can predispose the affected tooth to other dental disorders, such as wear and caries.^27^

### Amelogenesis Imperfecta

Amelogenesis imperfecta is a heterogeneous group of genetic conditions characterized by defects in the enamel formation of both deciduous and adult teeth.^67^ It can significantly impact the functionality of teeth, with enamel defects leading to compromised dental integrity. This may contribute to tooth loss and to challenges in grazing efficiency.^64,66^ Suspected Amelogenesis imperfecta was observed in 1% of 10-month-old Merino ewes and 37% of 20-month-old Dohne Merino ewes. Amelogenesis imperfecta can have considerable variation in the appearance of enamel defects. While it may appear as irregular depressions or pits on the enamel surface, with shallow pits of 1 to 2 mm on the incisors’ surface,^31,38^ other presentations include smooth hypoplastic variations, hypocalcified types where enamel thickness is normal but of poor quality and softness, and hypomature variants that are generally milder, often showing a snow-capped appearance with whiter incisor tips. These variations depend on the specific stage of amelogenesis that has been affected. The 10-month-old Merino ewes showed a much lower prevalence of Amelogenesis imperfecta compared to the 20-month-old Dohne Merinos, raising the possibility of hereditary factors influencing the occurrence of Amelogenesis imperfecta.

### Clinical Impacts

The ability of sheep to survive, maintain production, and experience positive welfare in diverse and often challenging environments depends on a functional dental apparatus that enables successful grazing and foraging.^16,47^ Loss of incisor function significantly reduces food intake per bite, affecting nutrition and productivity.^5^ Periodontitis is considered to be the primary cause of incisor loss in sheep,^15,39^ with other potential contributors, such as caries, receiving little attention. Structural dental anomalies such as Amelogenesis imperfecta, localized enamel hypoplasia, twinning anomalies, and talon cusps may create plaque traps and reduced resistance to acid dissolution and erosive wear,^68^ potentially predisposing affected sheep to caries and other dental diseases. Although these conditions may not directly cause functional issues (apart from Amelogenesis imperfecta, and localized enamel hypoplasia, which can cause sensitivity), they can significantly impact the structural integrity of the dental apparatus, increasing the risk of dental dysfunction over time. Direct research on pulp exposure in sheep is limited, studies in other species, including dogs and rats, indicate that dental pulp exposure can lead to discomfort or pain, resulting in changes in mastication, eating habits, and overall welfare.^69,70^ The parallels between species suggest that undiagnosed or untreated dental disorders, including pulp exposure and caries, may contribute to compromised dental function in sheep. Furthermore, given that these structural anomalies increase susceptibility to caries and other oral health issues in humans,^68^ it is plausible that similar implications may apply to sheep. Addressing these knowledge gaps through further research is essential for enhancing dental health and welfare management in sheep production systems.

## Conclusion

This study provides the first comprehensive exploration into various dental anomalies affecting sheep incisors, documenting conditions previously unreported in the species. Age differences were notable in patterns of incisor loss, with younger sheep showing more central incisor loss. In comparison, older sheep exhibited a more widespread distribution of missing teeth, suggesting progressive wear and health challenges for permanent incisors. Plaque accumulation also varied by age and tooth surface, with younger sheep displaying more variation, likely due to diverse environmental and dietary factors, contrasting with the more uniform patterns observed in older sheep. Caries, although rare, was documented, prompting questions about its development in sheep and about the role of anatomical features such as pits and fissures. The study identified enamel defects and genetic factors in conditions such as Amelogenesis imperfecta, especially in older sheep. Incisor anomalies such as gemination, talon cusp, and localized enamel hypoplasia were also noted. A more comprehensive understanding of the prevalence and types of dental conditions in Merino sheep will allow for more targeted management strategies, promoting better health and productivity across the industry.

## Materials

- Peak Hill Industries, New South Wales, Australia
- Bonida HD, MES, Keilor Park, Victoria, Australia

## References

[1] Holmes M Thomas R Hamerow H . Periodontal disease in sheep and cattle: understanding dental health in past animal populations. Int J Paleopathol. 2021;33(1):43–54. doi:10.1016/j.ijpp.2021.02.002 33647860

[2] de Arcaute MR Lacasta D González JM , et al. Management of risk factors associated with chronic oral lesions in sheep. Animals. 2020;10(9):1529. doi:10.3390/ani10091529 32872584 PMC7552339

[3] Silva NS Silveira JAS Lima DHS , et al. Epidemiological, clinical and pathological aspects of an outbreak of periodontitis in sheep. Pesq Vet Bras. 2016;36(11):1075–1080. doi:10.1590/S0100-736X2016001100003

[4] Baird AN Shipley CF . Oral-esophageal diseases. In: Pugh DG Baird AN Edmondson M Passler T , eds. Sheep, Goat, and Cervid Medicine. 3rd ed. Elsevier; 2021:51–62.

[5] Hongo A Zhang J Toukura Y Akimoto M . Changes in incisor dentition of sheep influence biting force. Grass Forage Sci. 2004;59:(3):293–297.

[6] Sykes AR Field AC Gunn RG . Effects of age and state of incisor dentition on body composition and lamb production of sheep grazing hill pastures. J Agric Sci. 1974;83(1):135–143. doi:10.1017/S0021859600047092

[7] Dove H Milne JA . An evaluation of the effects of incisor dentition and of age on the performance of lactating ewes and their lambs. Anim Sci. 1991;53(2):183–190. doi:10.1017/S0003356100020109

[8] Purser AF Wiener G West DM . Causes of variation in dental characters of Scottish blackface sheep in a hill flock, and relations to ewe performance. J Agric Sci. 1982;99(2):287–294. doi:10.1017/S0021859600030045

[9] Barnicoat C . Wear in sheep’s teeth. N Z J Sct Technol. 1957;38(Section A):583–632.

[10] Williams A . *Evaluation of Tooth Grinding as a Method for Improving Economic Performance in Flocks with Premature Incisor Tooth Loss (“broken Mouth”): Final Report, No. DAV 5*.; 1993.

[11] Dove H Freer M Foot JZ . The nutrition of grazing ewes during pregnancy and lactation: relationships between herbage, supplement and milk intakes, and ewe and lamb liveweight and body composition. Anim Prod Sci. 2018;58(7):1253. doi:10.1071/AN16541

[12] Bath JG Hogg RJ Edwards MSH . Merino wethers v. Crossbred ewes and lambs. Vic J Agric. 1965;63:251–258.

[13] McGregor BA Butler KL . The relationship between permanent incisor wear and mohair production and attributes in grazing adult angora goats. Small Rumin Res. 2011;100(1):37–43. doi:10.1016/j.smallrumres.2011.05.004

[14] Miles A Grigson C . Periodontal disease. In: Miles A Grigson C , eds. Colyer’s Variations and Diseases of the Teeth of Animals. Cambridge University Press; 1990:552–562.

[15] Spence J Aitchison G . Clinical aspects of dental disease in sheep. In Pract. 1986;8(4):128–135. doi:10.1136/inpract.8.4.128

[16] West D Bruere A Ridler A . The Sheep—Health, Disease and Production. 4th ed. Massey University Press; 2018.

[17] Floyd MR . The modified triadan system: nomenclature for veterinary dentistry. J Vet Dent. 1991;8(4):18–19. 1815632

[18] Cocquyt G Driessen B Simoens P . Variability in the eruption of the permanent incisor teeth in sheep. Vet Rec. 2005;157(20):619–623. doi:10.1136/vr.157.20.619 16284330

[19] Kyllar M Witter K . Prevalence of dental disorders in pet dogs. VetMed-Czech. 2005;11(50):496–505. doi:10.17221/5654-VETMED

[20] Kortegaard HE Eriksen T Baelum V . Screening for periodontal disease in research dogs—a methodology study. Acta Vet Scand. 2014;56:77. doi:10.1186/s13028-014-0077-8 PMC4240878 25407813

[21] Hennet P Servet E Salesse H Soulard Y . Evaluation of the Logan & Boyce plaque index for the study of dental plaque accumulation in dogs. Res Vet Sci. 2006;80(2):175–180. doi:10.1016/j.rvsc.2005.05.006 16229871

[22] Boy S Crossley D Steenkamp G . Developmental structural tooth defects in dogs—experience from veterinary dental referral practice and review of the literature. Front Vet Sci. 2016;3:9–9. doi:10.3389/fvets.2016.00009 26904551 PMC4744861

[23] Honma K Yamakawa M Yamauchi S Hosoy S . Statistical study on the occurrence of dental caries of domestic animals I. Horse. Jpn J Vet Res. 1962;10(1):31–36. doi:10.14943/jjvr.10.1.31

[24] Jackson K Kelty E Staszyk C Tennant M . Peripheral caries and disease of the periodontium in western Australian horses: an epidemiological, anatomical and histopathological assessment. Equine Vet J. 2019;51(5):617–624. doi:10.1111/evj.13084 30740768

[25] Lee L Reardon RJM Dixon PM . A post-mortem study on the prevalence of peripheral dental caries in Scottish horses. Equine Vet Educ. 2019;31(2):96–101. doi:10.1111/eve.12783

[26] Campello PL Borsanelli AC Agostinho SD , et al. Occurrence of periodontitis and dental wear in dairy goats. Small Rumin Res. 2019; 175(2):133–141. doi:10.1016/j.smallrumres.2019.05.004

[27] Kierdorf H Witzel C Upex B Dobney K Kierdorf U . Enamel hypoplasia in molars of sheep and goats, and its relationship to the pattern of tooth crown growth. J Anat. 2012;220(5):484–495. doi:10.1111/j.1469-7580.2012.01482.x 22352403 PMC3403278

[28] Ackermans NL Clauss M Winkler DE , et al. Root growth compensates for molar wear in adult goats (capra aegagrus hircus). J Exp Zool A Ecol Integr Physiol. 2019;331(2):139–148. doi:10.1002/jez.2248 30511369

[29] Stauffer JB Clauss M Müller DWH Hatt JM Ackermans NL . Testing inner-mesowear III on goats (Capra aegagrus hircus) fed experimental diets. Ann Zool Fennici. 2019;56(1-6):85–91. doi:10.5735/086.056.0108

[30] Fadden AN Poulsen KP Vanegas J Mecham J Bildfell R Stieger-Vanegas SM . Dental pathology in conventionally fed and pasture managed dairy cattle. Vet Rec. 2016;178(1):19–19. doi:10.1136/vr.103266 26700105

[31] West D . Dental disease of sheep. N Z Vet J. 2002;50(sup3):102–104. doi:10.1080/00480169.2002.36282 21838636

[32] Borsanelli AC Athayde FRF Agostinho SD Riggio MP Dutra IS . Dental biofilm and its ecological interrelationships in ovine periodontitis. J Med Microbiol. 2021;70(7):1–8. doi:10.1099/JMM.0.001396 34313584

[33] Bruere AN West DM Orr MB O’Callaghan MW . A syndrome of dental abnormalities of sheep: I. Clinical aspects on a commercial sheep farm in the Wairarapa. N Z Vet J. 1979; 27:152–158. doi:10.1080/00480169.1979.34632 291821

[34] Frisken KW Laws AJ Tagg JR Orr MB . Environmental influences on the progression of clinical and microbiological parameters of sheep periodontal disease. Res Vet Sci. 1989;46:147–152. 2784861

[35] Laws AJ Frisken KW Orr MB . A study of periodontal disease in sheep over a twelve month period. N Z Vet J. 1988;36(1):32–34. 16031430 10.1080/00480169.1988.35470

[36] Orr MB Chalmers M . A field study of the association between periodontal disease and body condition in sheep. N Z Vet J. 1988;36(4):171–172. doi:10.1080/00480169.1988.35524 16031484

[37] Wetselaar P Wetselaar-Glas MJM Katzer LD Ahlers MO . Diagnosing tooth wear, a new taxonomy based on the revised version of the tooth wear evaluation system (TWES 2.0). J Oral Rehabil. 2020;47(6):703–712. doi:10.1111/joor.12972 32274827 PMC7384115

[38] Crawford PJM Aldred M Bloch-Zupan A . Amelogenesis imperfecta. Orphanet J Rare Dis. 2007;2(17):1–11. doi:10.1186/1750-1172-2-17 17408482 PMC1853073

[39] Ridler AL West DM . Diseases of the oral cavity. In: Aitken ID ed. Diseases of Sheep. 4th ed. Massey University; 2007:148–155.

[40] Dachi SF Howell FV . A survey of 3,874 routine full-mouth radiographs. Oral Surg Oral Med Oral Pathol. 1961;14(10):1165–1169. doi:10.1016/0030-4220(61)90204-3 13883048

[41] Helsham RW . Some observations on the subject of roots of teeth retained in the jaws as a result of incomplete exodontia*. Aust Dent J. 1960;5(2):70–77. doi:10.1111/j.1834-7819.1960.tb03154.x

[42] Rustogi KN Curtis JP Volpe AR Kemp JH McCool JJ Korn LR . Refinement of the modified navy plaque Index to increase plaque scoring efficiency in gumline and interproximal tooth areas. J Clin Dent. 1992;3(Suppl C):C9–12. 1306676

[43] Turesky S Gilmore ND Glickman I . Reduced plaque formation by the chloromethyl analogue of victamine C. J Periodontol. 1970;41(1):41–43. doi:10.1902/jop.1970.41.1.41 5264376

[44] Estai M Kanagasingam Y Mehdizadeh M , et al. Mobile photographic screening for dental caries in children: diagnostic performance compared to unaided visual dental examination. J Public Health Dent. 2022;82(2):166–175. doi:10.1111/jphd.12443 33495989

[45] Smith BG Knight JK . An index for measuring the wear of teeth. Br Dent J. 1984;156(12):435–438. doi:10.1038/sj.bdj.4805394 6590081

[46] Cocquyt G Van den Broeck W Driessen B Simoens P . Variations of the canine teeth in sheep. Vlaams Diergeneeskd Tijdschr. 2003;72(5):332–339. https://www.researchgate.net/publication/257930332

[47] Tatara MR Szabelska A Krupski W , et al. Morphometric, densitometric and mechanical properties of mandibular deciduous teeth in 5-month-old Polish merino sheep. BMC Vet Res. 2014;10(45):1–6. doi:10.1186/1746-6148-10-45 24548814 PMC3936944

[48] Baker J Britt D . Dental calculus and periodontal disease in sheep. Vet Rec. 1984;115(16):411–412. doi:10.1136/vr.115.16.411 6506421

[49] Beg A Altaf G Garg S Anand M . Clinical study of pit and fissure morphology and its relationship with caries prevalence in young permanent first molars. J South Asian Assoc Pediatr Dent. 2019;2(2):56–60. doi:10.5005/jp-journals-10077-3032

[50] Al-Ramahi HM . Some incisor teeth problems in sheep in Al-diwanyia province. AL-Qadissiyha J Vet Med Sci. 2005;4:9–12.

[51] Amjadian S Safavi EA Moaddab SH . Prevalence of dental problems in 200 lamb or sheep carcasses (Iran). J Vet Res. 2018;22(6):452–456.

[52] Atkinson JA Jackson JM Lowery G Taylor GD Rogers HJ Vernazza CR . Community water fluoridation and the benefits for children. Dent Update. 2023;50(7):565–569. doi:10.12968/denu.2023.50.7.565

[53] Hattab FN . Double talon cusps on supernumerary tooth fused to maxillary central incisor: review of literature and report of case. J Clin Exp Dent. 2014;6(4):e400–e407. doi:10.4317/jced.51428 PMC4282909 25593664

[54] El Backly RM Kotry GS Moussa H . Multidisciplinary management of a fused maxillary incisor: case report with 5-year follow-up. Clin Case Rep. 2021;9(2):775–786. doi:10.1002/ccr3.3629 33598244 PMC7869352

[55] James EP Johns DA Johnson K Maroli RK . Management of geminated maxillary lateral incisor using cone beam computed tomography as a diagnostic tool. J Conserv Dent. 2014;17(3):293–296. doi:10.4103/0972-0707.131810 24944458 PMC4056406

[56] Mahendra L Govindarajan S Jayanandan M Shamsudeen SM Kumar N Madasamy R . Complete bilateral gemination of maxillary incisors with separate root canals. Case Rep Dent. 2014;2014:1–4. doi:10.1155/2014/425343 PMC4164315 25254121

[57] Sarpangala M . Variants of Talon Cusp (Dens Evaginatus). J. Clin. Diagn Res. 2017;11(1):ZJ01–ZJ02. doi:10.7860/JCDR/2017/24042.9207 PMC5324526 28274081

[58] Sharma G Nagpal A . Talon cusp: a prevalence study of its types in permanent dentition and report of a rare case of its association with fusion in mandibular incisor. J Oral Dis. 2014;2014:1–6. doi:10.1155/2014/595189

[59] Ozcelik B Atila B . Bilateral palatal talon cusps on permanent maxillary lateral incisors: a case report. Eur J Dent. 2011;5:113–116. doi:10.1055/s-0039-1698866 21228961 PMC3019756

[60] Salanitri S Seow WK . Developmental enamel defects in the primary dentition: aetiology and clinical management. Aust Dent J. 2013;58(2):133–140. doi:10.1111/adj.12039 23713631

[61] Guatelli-Steinberg D . Macroscopic and microscopic analyses of linear enamel hypoplasia in Plio-Pleistocene South African hominins with respect to aspects of enamel development and morphology. Am J Phys Anthropol. 2003;120(4):309–322. doi:10.1002/ajpa.10148 12627527

[62] Niemiec B Gawor J Nemec A , et al. World small animal veterinary association global dental guidelines. J Small Anim Pract. 2020;61(7):E36–E161. doi:10.1111/jsap.13132 32715504

[63] Orr M O’Callaghan M West D Bruere A . A syndrome of dental abnormalities of sheep. II. The pathology and radiology. N Z Vet J. 1979;27(12):276–278. 295108 10.1080/00480169.1979.34672

[64] Suckling G . Defects of enamel in sheep resulting from trauma during tooth development. J Dent Res. 1980;59(9):1541–1548. 6931141 10.1177/00220345800590092701

[65] Witter K . Mísek I. Time programme of the early tooth development in the domestic sheep (Ovis aries, ruminantia). Acta Vet Brno. 1999;68:3–8.

[66] Suckling G Elliott DC Thurley DC . The production of developmental defects of enamel in the incisor teeth of penned sheep resulting from induced parasitism. Arch Oral Biol. 1983;28(5):393–399. doi:10.1016/0003-9969(83)90134-6 6578757

[67] Smith CEL Poulter JA Antanaviciute A , et al. Amelogenesis imperfecta; genes, proteins, and pathways. Front Physiol. 2017;26(8):435. doi:10.3389/fphys.2017.00435 PMC5483479 28694781

[68] Reynolds L Dave M Tattar R Barry S . Inherited dental anomalies—part 1: enamel defects. Fac Dent J. 2024;15(3):98–106. doi:10.1308/rcsfdj.2024.31

[69] Gere I Dixon PM . Post mortem survey of peripheral dental caries in 510 Swedish horses. Equine Vet J. 2010;42(4):310–315. doi:10.1111/j.0425-1640.2009.00024.x 20525048

[70] Hernández SZ Negro VB du Puch G Saccomanno DM . Caries dentales en perros: nuestra experiencia. Rev Med Vet (B Aires). 2019;100(2):1–6.

